# Dynamic reprogramming of DNA methylation in SETD2-deregulated renal cell carcinoma

**DOI:** 10.18632/oncotarget.6481

**Published:** 2015-12-05

**Authors:** Rochelle L. Tiedemann, Ryan A. Hlady, Paul D. Hanavan, Douglas F. Lake, Raoul Tibes, Jeong-Heon Lee, Jeong-Hyeon Choi, Thai H. Ho, Keith D. Robertson

**Affiliations:** ^1^ Center for Epigenetics, Van Andel Research Institute, Grand Rapids, MI, USA; ^2^ Department of Molecular Pharmacology and Experimental Therapeutics and Mayo Clinic Comprehensive Cancer Center, Mayo Clinic, Rochester, MN, USA; ^3^ School of Life Sciences, Mayo Clinic Collaborative Research Building, Arizona State University, Scottsdale, AZ, USA; ^4^ Division of Hematology and Medical Oncology, Mayo Clinic, Scottsdale, AZ, USA; ^5^ Epigenomics Translational Program, Center for Individualized Medicine, Rochester, MN, USA; ^6^ Department of Biochemistry and Molecular Biology, Mayo Clinic, Rochester, MN, USA; ^7^ Department of Biostatistics, Georgia Regents University, Augusta, GA, USA

**Keywords:** DNA methylation, epigenetics, SETD2, histone methylation, renal cell cancer

## Abstract

Clear cell renal cell carcinomas (ccRCCs) harbor frequent mutations in epigenetic modifiers including SETD2, the H3K36me3 writer. We profiled DNA methylation (5mC) across the genome in cell line-based models of *SETD2* inactivation and *SETD2* mutant primary tumors because 5mC has been linked to H3K36me3 and is therapeutically targetable. SETD2 depleted cell line models (long-term and acute) exhibited a DNA hypermethylation phenotype coinciding with ectopic gains in H3K36me3 centered across intergenic regions adjacent to low expressing genes, which became upregulated upon dysregulation of the epigenome. Poised enhancers of developmental genes were prominent hypermethylation targets. *SETD2* mutant primary ccRCCs, papillary renal cell carcinomas, and lung adenocarcinomas all demonstrated a DNA hypermethylation phenotype that segregated tumors by SETD2 genotype and advanced grade. These findings collectively demonstrate that SETD2 mutations drive tumorigenesis by coordinated disruption of the epigenome and transcriptome,and they have important implications for future therapeutic strategies targeting chromatin regulator mutant tumors.

## INTRODUCTION

Cancer of the kidney and renal pelvis affects > 65,000 patients annually and ranks 8^th^ in causes of cancer death in the United States. The most common histologic subtype is clear cell renal cell cancer (ccRCC), which accounts for the majority of RCC-related deaths. Surgery remains the standard of care for patients with early stage tumors, however ∼30% of patients progress to distant metastases after surgery for localized disease. Despite some advances in systemic therapy, median survival drops to about two years after development of metastatic disease [1]. CcRCC differs from many tumor types in that it is characterized by frequent mutation of epigenetic regulators (dominated by SETD2 (10-15%), PBRM1 (33-45%), and BAP1 (15%)), while mutations in other common cancer gene pathways (e.g. RAS, BRAF, TP53, RB) are largely absent [2-5], and ccRCC is tightly linked to a distinct transcriptional signature due to inactivation of the *VHL* gene, which is mediated in part through deregulation of the epigenome [6]. These properties also make ccRCC an ideal tumor type to use as a model for determining how mutations in epigenetic regulator genes modulate tumor initiation and progression.

SETD2 is a ubiquitiously expressed SET domain-containing histone 3 lysine 36 trimethylase (H3K36me3) that interacts with elongating RNA pol II *via* the RNA pol II-associated factor complex (PAF1c), to recruit H3K36me3 to transcribing gene bodies [7-10]. SETD2 is the principle mediator of H3K36me3 and has little if any role in generating H3K36me1/me2 [11-13]. Functions for H3K36me3 include regulation of Pol II and nucleosome density across exons [2, 14], alternative splicing [15], and DNA repair [16, 17]. In ccRCC, biallelic *SETD2* inactivation is associated with reduced survival and earlier time to recurrence [18, 19]. Metastatic ccRCC displays markedly reduced H3K36me3 levels compared to matched primary ccRCCs [13]. These findings strongly suggest that *SETD2* mutations drive tumor progression, yet the underlying mechanism remains unknown.

Like H3K36me3, DNA methylation (5mC) is enriched across gene bodies [20] where it is positively linked to transcription [21] and regulates intragenic enhancer activity [22]. Four DNA methyltransferase family members, DNMT1, 3A, 3B, and 3L collectively establish and maintain genome-wide patterns of DNA methylation [23]. 5mC patterns in cancer are profoundly disrupted, with global hypomethylation affecting repetitive DNA and gene bodies accompanied by more focused promoter/CpG island (CGI)/CGI shore hypermethylation that silences the associated gene. Aberrant DNA methylation is sufficient to drive tumorigenesis in the absence of genetic mutations [24]. A direct link between DNA and H3K36 methylation was first revealed through *in vitro* binding studies, wherein recombinant Dnmt3a bound H3K36me2/me3-containing peptides and nucleosomes *via* its N-terminal PWWP domain [25] and subsequent chromatin interaction assays showed that H3K36me3 co-immunoprecipitates with Dnmt3b [26]. The PWWP domain is a moderately conserved motif in > 60 proteins, many of which associate with chromatin [27], that is now recognized as a reader domain for H3K36 methylation [15, 28].

The collective findings linking 5mC to H3K36me3 and *SETD2* mutations to ccRCC motivated us to examine their interplay in *SETD2* mutant tumors. Using cell line models we show that SETD2 loss-of-function induces global loss of H3K36me3, but also formation of ectopic H3K36me3. SETD2 inactivation also results in global redistribution of 5mC, with a predominance of hypermethylation events targeted to sites of ectopic H3K36me3, intergenic loci, and normal kidney poised enhancers. Functionally, global DNA hypermethylation events occur in large DMRs conserved across multiple tumor types with *SETD2* mutations and result in up-regulation of lowly expressed genes that collectively appear to drive cells toward a more undifferentiated state.

## RESULTS

### Validation of *SETD2* knockout (KO) 786-O cells as a model of *SETD2* mutated ccRCC

To generate a model to study the impact of *SETD2* mutations, we utilized the 786-O ccRCC cell line and targeted the *SETD2* locus for inactivation using zinc-finger nucleases (ZFNs). Two independent clones were isolated and characterized. In KO1 the ZFN-nuclease generated a 4 bp deletion and in KO2 an 11 bp deletion in *SETD2*, both causing frameshifts (Figure S1A). The two *SETD2* isogenic KO clones derived from parental 786-O ccRCC cells were validated by Sanger sequencing and cell line authentication (ATCC, data available upon request). Altered epigenetic phenotypes were highly consistent between the SETD2 KO1 and KO2 clones, as will be described.

Since one of our goals was to determine the impact of SETD2 loss-of function on 5mC patterns, we first examined the impact of this mutation on components of the DNA methylation machinery by RNA-seq and qRT-PCR (Figure S1B). In 786-O parental cells, DNMT1 was the most highly expressed DNMT (Figure S1B, left axis) consistent with its role as the maintenance methyltransferase. Expression of the *de novo* methyltransferases was low in parental 786-O cells; DNMT3L was undetectable (Figure S1B, left axis). Inactivation of SETD2 in 786-O cells down-regulated DNMT1 and up-regulated DNMT3B to some extent; DNMT3A and DNMT3L expression did not change (Figure S1B, right axis). Expression of the TETs in parental 786-O was variable (Figure S1B, left axis). SETD2 inactivation resulted in down-regulation of TET1 and up-regulation of TET3 (Figure S1B, right axis). Taken together, there were no consistent changes in expression of DNA methylation machinery components that would likely account for global changes in 5mC between parental and SETD2 KO 786-O clones. Since the DNMTs and TETs play important roles in development, we also assayed expression of pluripotency and germ layer markers upon SETD2 inactivation (Figure S1C). SETD2 KO altered expression of these markers with up-regulation of pluripotency markers and variable changes in expression among germ layer markers (Figure S1C). Subunits of the PAF complex, which interacts with both SETD2 and the DNMT3s [8, 29] remained constant (Figure S1C).

### Loss of SETD2 induces redistribution of H3K36me3

**Figure 1:**
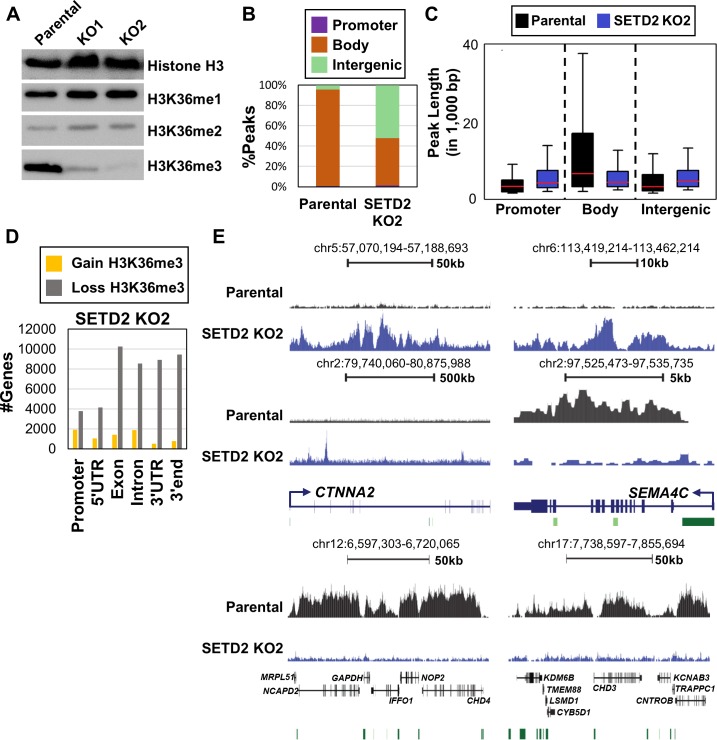
Reduction and redistribution of H3K36me3 upon inactivation of *SETD2* in 786-O ccRCC cells **A.** Western blot of H3K36 methylation in parental 786-O cells and two independent *SETD2* KO clones. Histone H3 is a loading control. **B.** Distribution of H3K36me3 ChIP-seq peaks genome-wide in 786-O ccRCC cells. **C.** Box plots for H3K36me3 peak length across genomic features. Mean line is represented in red. **D.** Number of genes with ≥ 2-fold-change in H3K36me3 level in SETD2 KO clone 2. **E.** Representative browser shots demonstrating loss and gain of H3K36me3 in *SETD2* KO2 clone. Top panel represents intergenic regions that gain H3K36me3. Bent arrows = TSS, Green bars = CpG islands. See also Figure S1, S2.

SETD2 KO in 786-O cells resulted in global reduction of H3K36me3 with little effect on total H3K36me1 and H3K36me2 (Figure 1A). However, H3K36me3 was not completely depleted upon *SETD2* inactivation using moderate exposures in the western blotting. We next performed chromatin immunoprecipitation sequencing (ChIP-seq) of H3K36me3 in 786-O parental and *SETD2* KO clones to map its genome-wide distribution. Consistent with reduction of H3K36me3 upon *SETD2* inactivation (Figure 1A), coverage from H3K36me3 ChIP-seq (relative to the total bp covered by sequence reads) decreased to 17.6% and 15.2% for clone 1 and clone 2, respectively, from 31.4% observed in parental 786-O cells. As expected, the majority of H3K36me3 peaks observed in parental 786-O cells were enriched within gene bodies (Figure 1B). In the SETD2 KO clones, however, a marked redistribution of the remaining H3K36me3 was observed, with gains of this mark primarily occurring in intergenic regions (Figure 1B). Loss of H3K36me3 also occurred upon *SETD2* inactivation, as would be expected, with nearly 40% fewer H3K36me3 peaks observed in gene bodies of the KOs relative to parental 786-O (Figure 1B). Indeed, the length of peaks across gene bodies was reduced among the SETD2 KO clones relative to the parental 786-O cells, while the peak length in intergenic regions increased with *SETD2* inactivation (Figure 1C). To evaluate the possibility of non-specific binding of the H3K36me3 ChIP antibody, we performed dot blotting with peptides containing other histone modifications and determined that the antibody had high specificity for H3K36me3 and no cross reactivity with H3K36me2 or H3K36me1 (Figure S2A). We next assayed differential enrichment of H3K36me3 by SICER-DF analysis [30] among the 786-O parental and *SETD2* KO clones. Loss of H3K36me3 in *SETD2* KO clones occurred predominately in gene bodies (Figure 1D, Figure S2B). However, a small number of genes gained H3K36me3 upon inactivation of SETD2 with no marked enrichment in any particular feature (Figure 1D, Figure S2B). Regions of the genome that gained and lost H3K36me3 were highly conserved among both independent *SETD2* KO clones (Figure 1E, Figure S2C). As gains in H3K36me3 were unexpected, we validated our H3K36me3 ChIP-seq with locus-specific ChIP-qPCR (Figure S2D). Overall, we observed predominantly reduction in H3K36me3 as a result of *SETD2* KO in 786-O cells, but also gains of H3K36me3 over gene bodies and intergenic regions.

### SETD2 inactivation results in DNA hypermethylation that coincides with regions of ectopic H3K36me3

**Figure 2:**
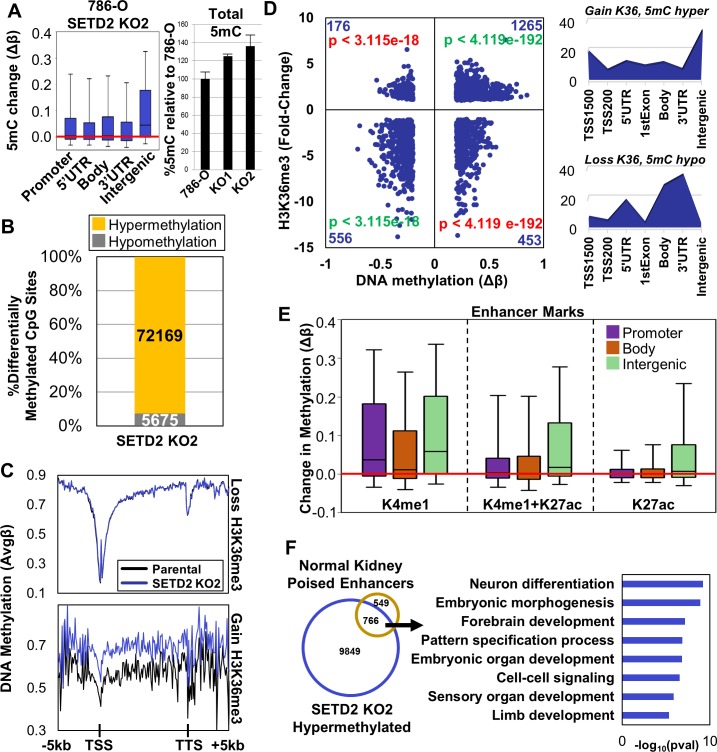
Distribution of DNA methylation genome-wide and integration with H3K36me3 under SETD2 loss-of-function conditions **A.** Left. Box plots representing all Δβ-values (change in 5mC) in the indicated features. Right. Quantification of total 5mC levels in genomic DNA by LC-MS/MS. Each sample was run in duplicate and values are the average and standard deviation relative to parental 786-O set at 100%. The 5mC increases in both KO1 and KO2, relative to 786-O are significant (two-sample *t* test). Actual average 5mC/10^6^ dC values are: 786-O 42,083, KO1 52,581, and KO2 57,367. **B.** Percentage of differentially methylated CpG sites (|Δβ| ≥ 0.2) classified by methylation change upon SETD2 KO (hypo/hypermethylation). **C.** Spatial distribution plots of DNA methylation across intragenic regions derived from average β-values stratified by loss or gain in H3K36me3 upon SETD2 KO. **D.** Left: Scatter plot of differentially methylated CpGs (|Δβ| ≥ 0.2) within peaks of H3K36me3 in 786-O cells indicating change in methylation (x-axis) *versus* change in H3K36me3 (y-axis). Number of CpGs is provided in the corner of each quadrant along with significance of 5mC/H3K36me3 overlap. Green = significant overlap; Red = significant exclusion. Right: Genomic feature enrichment plots normalized to the number of CpGs present in each feature on the 450K array for categories with significant overlap. **E.** Box plots representing the change in methylation of CpGs in peaks with the specified enhancer marks stratified by genomic feature. Enhancer mark annotations are derived from normal adult human kidney H3K27ac and H3K4me1 ChIP-seq from the Epigenome Roadmap. **F.** Left: Venn diagram of genes hypermethylated upon SETD2 KO and marked exclusively by H3K4me1 in adult human kidney. Right: Biological process ontology of the overlapping gene set. See also Figure S3.

Since H3K36me3 and 5mC overlap significantly in their genome-wide distribution [21, 26, 30, 31] we next assayed DNA methylation patterns in the 786-O isogenic clones using the Illumina HumanMethylation450 BeadChip (450K array). Globally, DNA hypermethylation was observed in both *SETD2* KO clones at all genomic features, but particularly intergenic regions (Figure 2A-left, Figure S3A). Quantification of total genomic 5mC content by LC-MS/MS [32] confirmed this observation, revealing that SETD2 inactivation resulted in > 20% increase in total 5mC in both SETD2 KO clones (Figure 2A-right). Analysis of the most differentially methylated CpGs (|Δβ|≥0.2) from the 450K array revealed that greater than 80% of differential methylation upon *SETD2* inactivation was in the direction of hypermethylation (Figure 2B, Figure S3B). DNA hypermethylation was focused primarily in intergenic regions while hypomethylation was enriched at gene termini where both H3K36me3 and 5mC peak under normal conditions (Figure S3C). Independent MeDIP-qPCR analysis validated the elevated 5mC events identified by 450K array (*CTNNA2, AJAP1,* and *SLIT2*) (Figure S3D). Additionally, we included in our MeDIP-qPCR confirmation an intergenic region on chromosome 5 that was validated for ectopic H3K36me3 (Figure S3D) as the 450K array does not provide coverage of this locus. Although subtle (most likely due to low CpG density of this region), hypermethylation of this intergenic region was observed in both *SETD2* KO clones (Figure S3D).

Next, we integrated the genome-wide distribution of DNA methylation and H3K36me3 in the isogenic 786-O cells to determine if they were coordinated. Genes that lost H3K36me3 upon *SETD2* KO did not display alterations in 5mC (Figure 2C, S3E top panel), rather DNA hypermethylation occurred at loci that acquired ectopic H3K36me3 (bottom panels in Figure 2C, Figure S3E). To further investigate the effect that H3K36me3 distribution had on 5mC, we assigned differentially methylated CpGs to categories based on occurrence with differential H3K36me3 peaks (Figure 2D, Figure S3F). Hypermethylated CpGs significantly overlapped with regions that gained H3K36me3 (Figure 2D, Figure S3F) with particular enrichment in intergenic regions (Figure 2D, Figure S3F, right). Hypomethylated CpGs significantly coincided with regions losing H3K36me3 at gene termini (Figure 2D, Figure S3F, right). Contrary to the observation that loss of H3K36me3 does not influence global 5mC distribution, our focused analysis reveals that a subset of gene termini do in fact require H3K36me3 for proper establishment of 5mC, suggesting that the interplay between H3K36me3 and 5mC differs within the gene body domain or can be influenced by other processes (e.g. 3′-end definition *versus* elongation or splicing).

### Poised enhancers in normal adult kidney are targeted for DNA hypermethylation and ectopic H3K36me3 in ccRCC

H3K4me1 is localized to both poised and active enhancers, while H3K27ac marks active enhancers [33]. Active enhancers are typically devoid of 5mC as these regions are hotspots for transcription factor binding [34]. To investigate the epigenetic regulation of enhancers in *SETD2* mutated ccRCC, we integrated ChIP-seq data for H3K4me1 and H3K27ac from normal adult human kidney (Epigenome Roadmap) with our 5mC and H3K36me3 profiles for 786-O parental and *SETD2* KO cells. We observed co-occurrence of hypermethylation in regions marked exclusively by H3K4me1 genome-wide, while regions marked with H3K27ac displayed hypermethylation in intergenic regions only (Figure 2E). Overlap analysis of the most differentially methylated CpGs in 786-O *SETD2* KOs revealed significant enrichment of hypermethylated CpGs at regions marked by H3K4me1 in normal adult kidney and exclusion of differential methylation at H3K27ac-marked regions (Figure S3G). Next, we classified genes from normal adult kidney marked with K4me1 only, K4me1+K27ac, or K27ac only (Figure S3H). Genes containing all enhancer marks were also determined (termed “All classes”). Expression of genes in normal kidney associated with the different groups of enhancer marks in a manner consistent with their reported functionality; genes marked by K4me1 alone (“poised” enhancers) demonstrated low expression and genes marked with K27ac exhibited higher expression (Figure S3I). Genes marked exclusively by H3K4me1 in normal adult kidney significantly overlapped with genes targeted for hypermethylation in 786-O *SETD2* KO (*p*val < 2.089e-07), and were enriched for developmental processes (Figure 2F). Finally, we determined the differential H3K36me3 status of the normal adult kidney enhancer classified genes in our 786-O *SETD2* KO cells. Genes marked exclusively by H3K4me1 in normal adult kidney demonstrated a broad range of differential H3K36me3 in 786-O *SETD2* KO clones (including ectopic gains), while genes marked with H3K27ac in normal kidney overwhelming lost H3K36me3 (Figure S3J). The mechanism by which poised enhancers are targeted for aberrant epigenomic regulation such as gains in H3K36me3 and 5mC remains unclear, but enhancers linked to genes regulating developmental processes are a major target of this effect. This finding is also consistent with our RT-PCR data showing up-regulation of pluripotency genes and differential effects on germ layer markers upon *SETD2* inactivation (Figure S1C).

### Reconfiguration of 5mC and H3K36me3 by SETD2 KO influences gene expression

**Figure 3:**
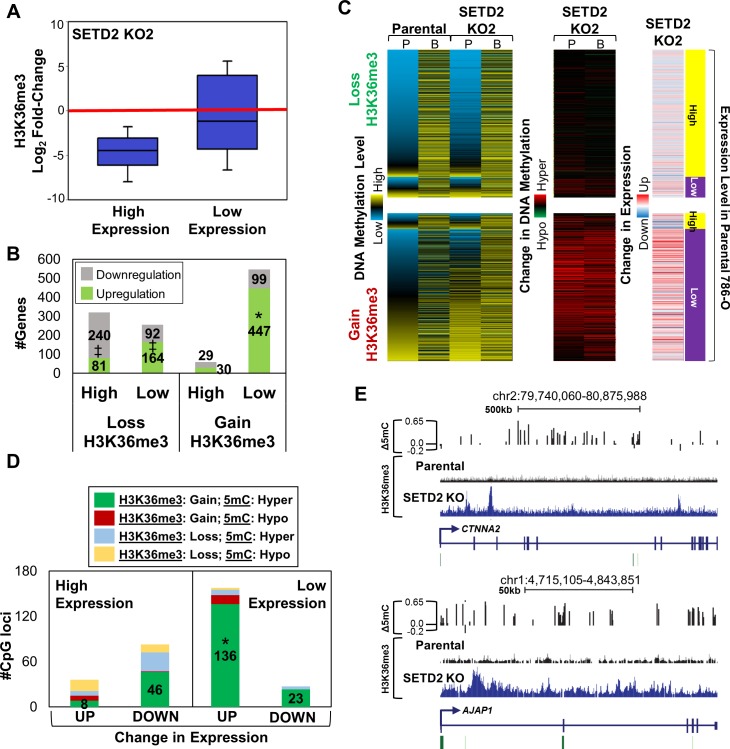
Redistribution of H3K36me3 with SETD2 inactivation is linked to changes in expression **A.** Box plot showing the fold-change in H3K36me3 relative to parental 786-O cells at high and low expressing genes based on RPKM values. **B.** Number of genes up-/down-regulated (≥ 2 fold change) in each expression tier (high/low) classified by whether the gene demonstrated loss or gain in H3K36me3 upon SETD2 KO. * = overlap (*p*val < E-50), ‡ = exclusion (*p*val < E-50) **C.** Heatmaps demonstrating the average DNA methylation level, change in methylation, and overall expression change for genes that lose/gain H3K36me3. 5mC level and the change in 5mC for CpGs (450K array) in genes that lose (top) or gain (bottom) H3K36me3 were averaged based on genomic location (*P* = promoter, B = body). Each row represents a gene. **D.** Number of CpGs showing the indicated change in H3K36me3 and 5mC stratified by expression tier and change in expression. * = overlap (*p*val < E-10) **E.** Browser shots demonstrating DNA hypermethylation and gain of H3K36me3 across two genomic loci. Bent arrows = TSS; Green bars = CpG islands. See also Figure S4.

Since DNA methylation, H3K36me3, and enhancer elements all play pivotal roles in gene regulation, we next examined the relationship between *SETD2* KO-associated epigenome reconfiguration and changes in gene expression. First, we stratified gene expression in parental 786-O cells into two expression tiers, high and low (including genes with no expression), by RPKM values. Next, we determined the fold-change in expression for both *SETD2* KO clones relative to parental cells. Overall, most differential expression (≥ 2 fold-change) occurs at genes belonging to the low expression tier, with a majority of differentially expressed genes being up-regulated upon SETD2 inactivation (Figure S4A). Conversely, genes within the high expression tier were typically down-regulated (Figure S4A). The change in H3K36me3 among genes stratified by expression tier was then determined. H3K36me3 loss induced by *SETD2* KO occurred at high expressing genes, while low expressing genes tended to gain ectopic H3K36me3 (Figure 3A, Figure S4B). Up-regulated genes from the low expression tier significantly overlapped with genes that gained H3K36me3 (*p*val < E-50), while genes that lost H3K36me3 were not enriched for differential gene expression (Figure 3B, Figure S4C). Integration of DNA methylation level and how it changed with *SETD2* KO revealed that genes undergoing loss of H3K36me3 do not sustain changes in 5mC or expression, and that these genes have the typical methylation profile of high expressing genes (low promoter 5mC, high gene body 5mC, Figure 3C, top). Filtering for genes in the high expression tier that lose H3K36me3 revealed the extent to which DNA methylation remains the same with *SETD2* KO (Figure S4D, top). Genes that gain H3K36me3, however, show marked changes in both 5mC and expression, with hypermethylation across all regions of the gene and elevated expression (Figure 3C, bottom). Indeed, evaluation of overall 5mC at genes within the low expression tier that gain H3K36me3 reveals hypermethylation in both *SETD2* KO clones (Figure S4D). Ontology analysis of genes that gain H3K36me3, 5mC, and expression demonstrate enrichment for processes involved in cell adhesion, signaling, and development (Figure S4E). To determine if differential methylation at base-pair resolution correlates with changes in H3K36me3 status and gene expression, we integrated differential expression with the categories described previously in Figure 2D. Significant overlap of genes changing in expression occurred only with hypermethylated CpGs in regions of increased H3K36me3 (Figure 3D, Figure S4F) indicating that gains, but not losses, of H3K36me3 specifically influence gene expression upon SETD2 inactivation. We validated up-regulation of genes that gain H3K36me3 and 5mC (Figure 3E) by qRT-PCR (Figure S4G). Finally, as epigenetic regulation of enhancer elements also influences gene expression, we determined if genes linked to a particular combination of enhancer marks (as in Figure S3H) were enriched for expression changes with *SETD2* KO. Indeed, genes marked exclusively by H3K4me1 were significantly enriched for up-regulation (Figure S4H), the same class that displayed enrichment for H3K36me3 gains (Figure S3J) and DNA hypermethylation (Figure 2E). Taken together, these results indicate that loss of SETD2 function induces marked redistribution of H3K36me3 and 5mC that positively influences expression of low expressing genes.

### DNA hypermethylation induced by SETD2 KO occurs over large regions of the genome

**Figure 4:**
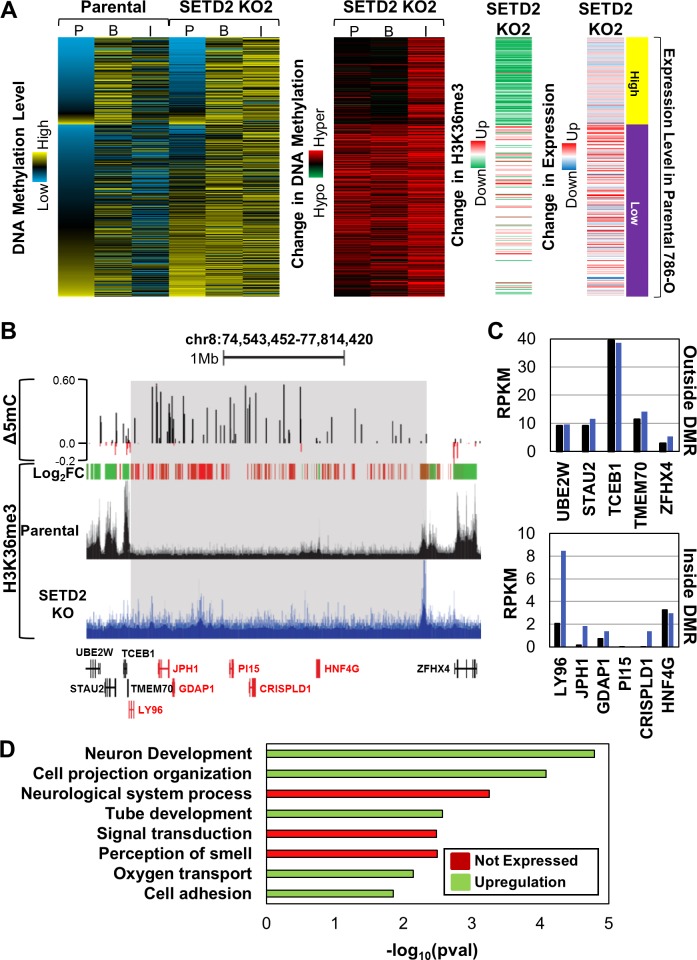
SETD2 loss-of-function induces ectopic H3K36me3 and DNA hypermethylation coordinated across large regions of the genome **A.** Heatmaps demonstrating the average 5mC level, change in 5mC, change in H3K36me3, and overall expression change for genes adjacent to hypermethylated (Δβ≥0.2) intergenic CpGs. 5mC level and change in 5mC for CpGs (450K array) in genes adjacent to hypermethylated intergenic CpG loci were averaged based on genomic location (*P* = promoter, B = body, I = intergenic). Each row represents a gene. **B.** Browser shot of a differentially methylated region (DMR) in which DNA hypermethylation, H3K36me3 gains, and up-regulation of gene expression occurs. DMR is highlighted in gray. Log_2_FC = Log_2_ fold-change in H3K36me3 where red indicates gains in H3K36me3 and green indicates loss of H3K36me3. Genes within the DMR are highlighted in red. **C.** Expression by RNA-seq of genes outside (top) and inside (bottom) the DMR. **D.** Ontology analysis of genes located in DMRs that are conserved between both SETD2 KO clones. Terms are highlighted by expression change of the genes that contribute. See also Figure S5.

Since a large proportion of differential hypermethylation occurred at intergenic regions with SETD2 inactivation (Figure 2A, Figure S3C), we next evaluated whether these were sporadic or coordinated events. Low expressing genes adjacent to hypermethylated intergenic CpGs were hypermethylated across both promoter and gene body regions (Figure 4A). Notably, a number of the genes demonstrated elevated H3K36me3 and expression (Figure 4A). High expressing genes adjacent to hypermethylated intergenic CpGs, in contrast, did not change their 5mC. Differentially methylated regions (DMRs) are defined as contiguous regions of the genome that undergo conserved changes in DNA methylation. Since genes adjacent to hypermethylated intergenic CpG sites also display hypermethylation, we assayed the SETD2 KO clones for DMRs (defined as eight contiguous CpGs with Δβ≥0.2) (Figure S5A). Eighty percent of identified DMRs from one *SETD2* KO clone were conserved in the other *SETD2* KO clone, indicating that these loci are consistently targeted for hypermethylation with SETD2 inactivation (Figure S5A). DMRs occurred predominately in intergenic regions (Figure S5B), coincided with large domains that gained H3K36me3 (Figure 4B) (*p*val < 2.65E-49), and genes within the DMR were typically up-regulated as a result (Figure 4C). In addition, a significant proportion of genes within DMRs are marked by H3K4me1 in normal adult kidney (*p*val < 5.75E-10). Finally, almost all genes within DMRs are low expression tier genes, and typically are not expressed or are up-regulated by *SETD2* KO (Figure S5C). Ontology analysis revealed enrichment for biological processes involved in development (likely a reflection of the genes marked previously by H3K4me1, Figure 2F), cell adhesion, and signal transduction (Figure 4D).

### SETD2 siRNA knockdown (KD) induces DNA hypermethylation in NCCIT embryonic carcinoma cells

**Figure 5:**
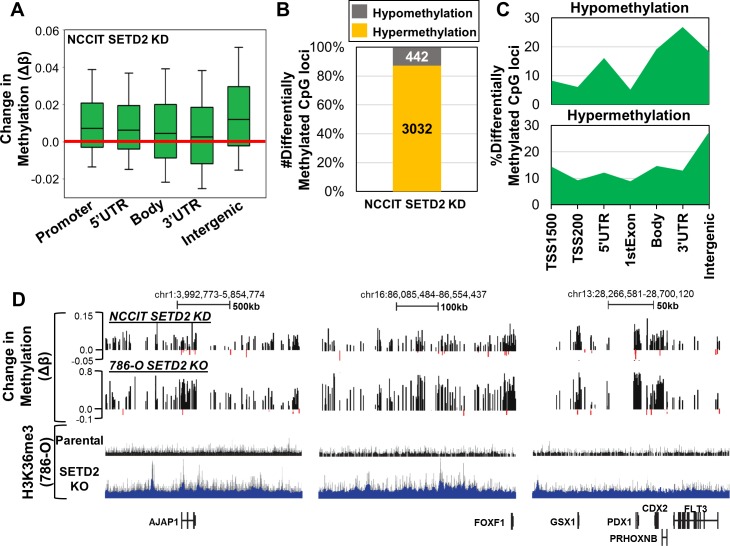
Acute depletion of SETD2 by siRNA in NCCIT embryonic carcinoma cells recapitulates DNA methylation changes observed in SETD2 KO 786-O cells **A.** Box plots representing all Δβ-values in the indicated genomic features. **B.** Percentage of differentially methylated CpG sites (|Δβ| ≥ 0.1) classified by methylation change upon SETD2 siKD (hypo/hypermethylation). **C.** Normalized distribution of significantly differentially methylated CpG sites by genomic feature. **D.** Representative browser shots demonstrating conservation of hypermethylation between NCCIT SETD2 siKD and 786-O SETD2 KO. H3K36me3 786-O ChIP-seq tracks are provided as a reference for H3K36me3 status across the regions. See also Figure S6.

To determine if DNA hypermethylation is a common phenotype induced by SETD2 loss-of-function outside the context of an RCC background, we acutely depleted SETD2 in NCCIT embryonic carcinoma cells using siRNA as we have done previously [31]. Total H3K36me3 was decreased upon siKD of SETD2 in NCCIT cells (Figure S6A) and did not significantly alter expression of housekeeping genes, epigenetic modifiers, and PAF complex subunits (Figure S6B). Pluripotency genes and germ layer markers were differentially expressed with SETD2 siKD (Figure S6B), similar to the changes observed in 786-O *SETD2* KO cells (Figure S1C). Next we assayed genome-wide 5mC patterns with the 450K array. Like 786-O *SETD2* KO cells, regional analysis of 5mC changes revealed hypermethylation occurring predominately in intergenic regions (Figure 5A). Analysis of the most differentially methylated CpGs (|Δβ|≥0.1) showed that > 85% of CpGs became hypermethylated upon SETD2 siKD (Figure 5B). Enrichment analysis of differentially methylated CpGs demonstrated DNA hypomethylation occurring predominately at gene termini, while hypermethylation events were enriched in intergenic regions (Figure 5C), patterns similar to those observed in 786-O *SETD2* KO cells (Figure S3C). Hypermethylation of promoters and gene bodies significantly overlapped between SETD2 siKD and *SETD2* KO cell models, while hypomethylation events overlapped only in gene bodies (Figure S6C). Finally, DNA hypermethylation induced by SETD2 siKD in NCCIT cells occurred at regions of the genome conserved with those observed in 786-O *SETD2* KO cells (Figure 5D). These were also regions that demonstrated ectopic H3K36me3 in the 786-O *SETD2* KO cells (Figure 5D). Taken together, these results show that DNA hypermethylation arising from SETD2 loss-of-function is conserved across cell types and occurs with both acute and long-term functional inactivation of SETD2.

### SETD2 mutant primary ccRCC manifests DNA hypermethylation consistent with cell line models

**Figure 6:**
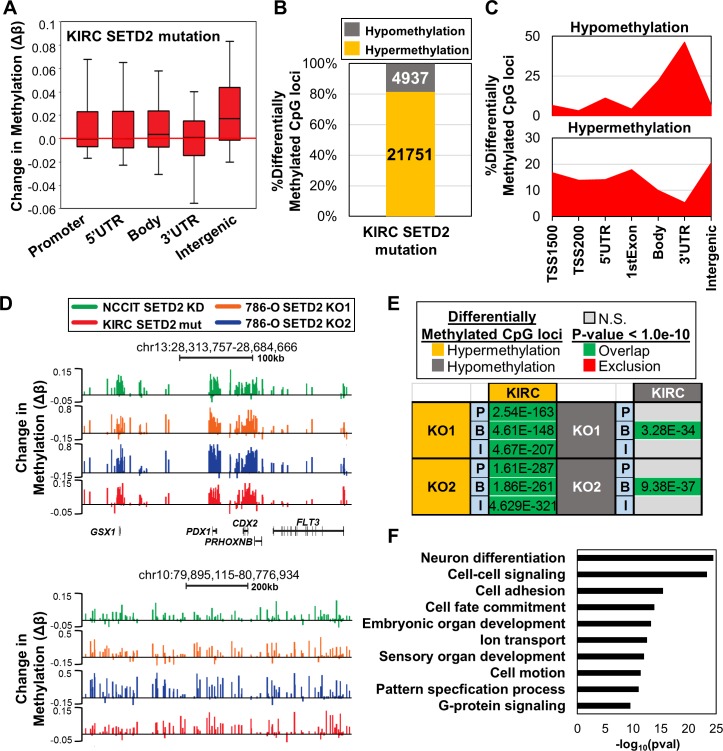
Epigenetic changes in SETD2 inactivation cell line models are recapitulated in *SETD2* mutated primary ccRCCs **A.** Box plots representing all Δβ-values in the indicated features in *SETD2* mutant ccRCC from the TCGA KIRC dataset. **B.** Percentage of differentially methylated CpG sites (|Δβ| ≥ 0.1) classified by 5mC change in *SETD2* mutated relative to WT ccRCC. **C.** Normalized distribution of significantly differentially methylated CpGs by genomic feature. **D.** Browser shots of regions that demonstrate consistent hypermethylation across all SETD2 altered conditions studied. Bottom region is an example of hypermethylation across an intergenic region. **E.** Overlap analysis of differentially methylated CpGs between 786-O SETD2 KO (|Δβ| ≥ 0.2) and *SETD2* mutated ccRCC (|Δβ| ≥ 0.1) across different genomic features. *P* = Promoter; B = Gene Body; I = Intergenic. Fisher's Exact Test was used to determine significance. **F.** Biological process ontology analysis (DAVID) of overlapping hypermethylated genes among 786-O SETD2 KO samples and *SETD2* mutated ccRCC. See also Figure S7.

After identifying epigenetic patterns conserved between different cell lines resulting from depletion of SETD2, we next determined if these alterations in 5mC occur in primary ccRCCs with *SETD2* mutations. Since > 90% of ccRCCs have *SETD2* LOH, but evidence that monoallelic loss of *SETD2* impacts global levels of H3K36me3 is lacking [13], we identified tumor samples from the Cancer Genome Atlas (TCGA) KIRC dataset with biallelic inactivation from copy number loss and concurrent *SETD2* mutation (*n* = 29) and compared these samples to KIRC tumors with no evidence of *SETD2* mutation or LOH (*n* = 20). To facilitate comparison with our *SETD2* KO cell lines, only KIRC samples with available 450K array data were used. Consistent with genome-wide changes in 5mC observed in both of our SETD2 loss-of-function cell line models, hypermethylation in the *SETD2* mutant primary ccRCCs occurred specifically at intergenic regions (Figure 6A). Focusing on the most differentially methylated CpGs (|Δβ|≥0.1) revealed that > 80% of these loci sustained hypermethylation (Figure 6B). Enrichment profiles for differentially methylated CpGs were also consistent with those observed in the cell line models, with hypomethylation events enriched at gene termini and hypermethylation events enriched at intergenic loci (Figure 6C). Indeed, hypermethylated DMRs conserved among SETD2 inactivated cell lines and primary tumors were identified, illustrating the reproducibility of DNA hypermethylation that accompanies loss of SETD2 function (Figure 6D). The overlap of hypermethylated CpGs among all genomic features, and of hypomethylated CpGs specifically across gene bodies between *SETD2* mutant cell lines and primary tumors was significant (Figure 6E). Ontology analysis of hypermethylated genes that overlap among 786-O *SETD2* KOs and *SETD2* mutated ccRCC tumors (Figure S7A) revealed enrichment of similar biological process terms, including developmental-related, cell adhesion, and transport (Figure 6F). A significant proportion of the overlapping genes were also marked exclusively by H3K4me1 in adult human kidney (*p*val < 1E-100), indicating that poised enhancers are targeted for aberrant epigenetic regulation in *SETD2* mutant primary tumors.

To determine if changes in H3K36me3 distribution were also conserved between our cell line model and primary *SETD2* mutant ccRCCs, we examined ChIP-seq data derived from two metastatic primary ccRCCs, one harboring WT *SETD2* and one with biallelic *SETD2* loss [13]. Locations that lost H3K36me3 under SETD2 inactivation conditions were highly conserved between cell lines and primary tumors (Figure S7B). Ectopic gains in H3K36me3, which occur less frequently than losses of H3K36me3, were also conserved (Figure S7B), but excluded from the *SETD2* WT tumor sample. Indeed, loci that consistently gain H3K36me3 and 5mC were identified among the cell lines and *SETD2* mutated primary ccRCC, but not the *SETD2* WT tumor (Figure S7C). Next, we stratified gene expression from the KIRC TCGA normal kidney samples into high and low expression tiers, and evaluated their 5mC levels. Consistent with the cell lines, hypermethylation in *SETD2* mutant KIRC ccRCCs was focused primarily on genes within the low expression tier, and hypomethylation on genes in the high expression tier (Figure S7D). Finally, we examined 5mC levels in *SETD2* WT versus mutant ccRCCs stratified by expression tier. Although subtle, hypermethylation in the *SETD2* mutated ccRCCs was observed at genes in the low expression tier, while genes in the high expression tier maintained their DNA methylation (Figure S7E). Taken together, results from the 786-O *SETD2* KO cells were highly predictive of epigenetic phenotypes that occur as a result of *SETD2* mutation in primary ccRCC.

### SETD2 loss-of-function mutations induce DNA hypermethylation in other tumor types

**Figure 7:**
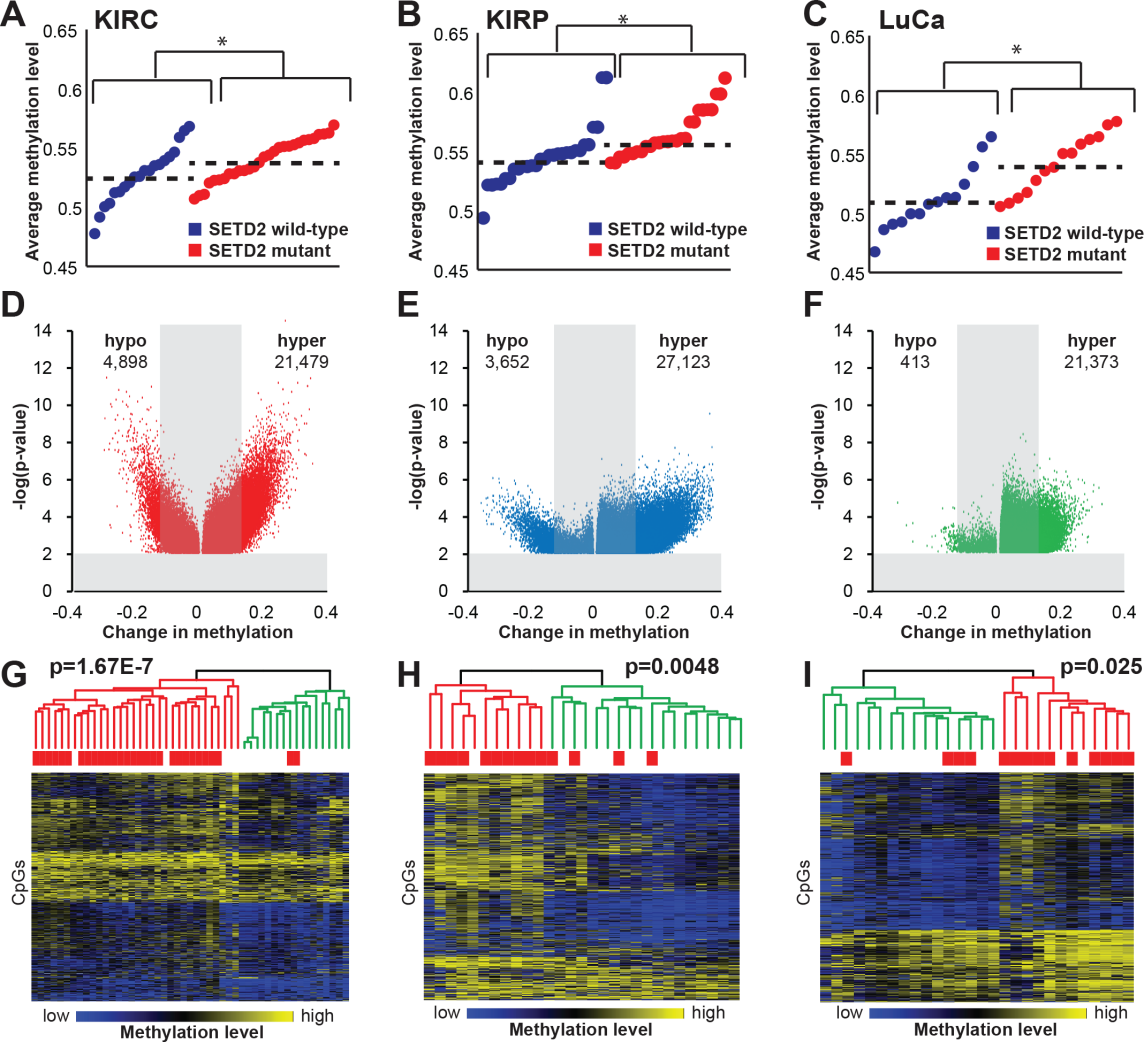
*SETD2* mutation is associated with a hypermethylation phenotype across multiple tumor types The average methylation value for each *SETD2* wild-type (blue) and mutant (red) tumor, as measured by the Infinium 450K for **A.** ccRCC (KIRC), **B.** papillary renal cell carcinoma (KIRP), and **C.** lung adenocarcinoma (LuCa). Significance between wild-type and mutant tumors is denoted by (*), *p* < 0.05. **D**.-**F**. Volcano plots presenting the number of hypo- and hypermethylated CpGs relative to their significance (−log p-value). **G.**-**I.** Heatmaps depicting unsupervised hierarchical clustering of the top 10,000 most variable CpGs in ccRCC, pRCC, and LuCa. A color bar is shown with low methylation in blue, intermediate in black, and high methylation in yellow. The two major dendrogram clusters are colored in red and green. Chi-square testing was used to determine statistical significance between dendrogram clusters. See also Figure S8.

As global DNA hypermethylation was consistently induced in our models of SETD2 inactivation and in primary *SETD2* mutant ccRCCs, we next investigated whether inactivating *SETD2* mutations in other cancer types are associated with a hypermethylation signature. We identified samples from the TCGA kidney renal papillary cell carcinoma (KIRP) and lung adenocarcinoma (LuCa) data collections that harbored *SETD2* mutations (two tumor types with appreciable numbers of *SETD2* mutant tumors in TCGA datasets) and analyzed them alongside the KIRC dataset [35]. *SETD2* mutations were significantly associated with global DNA hypermethylation in all three tumor types (Figure 7A-7C). Closer examination of 5mC profiles revealed that hypermethylation events in KIRC, KIRP, and LuCa were more frequent in number, magnitude (change in 5mC), and significance (p-value) in *SETD2* mutant tumors relative to the wild-type counterparts (Figure 7D-7F). Unsupervised hierarchical clustering of the top 10,000 most variably methylated CpGs from the 786-O *SETD2* KO samples and the KIRC dataset segregated samples based on *SETD2* genotype (Figure S8A). We next performed unsupervised hierarchical clustering based on the 10,000 most variable CpGs on the 450K array in each tumor type (Figure 7G-7I), revealing two major clusters based on 5mC profiles; one cluster dominated by hypermethylation that significantly coincided with high prevalence of *SETD2* mutation (KIRC, *p* = 1.67E-7; KIRP, *p* = 0.0048; LuCa, *p* = 0.025). Specifically, 86%, 83%, and 79% of KIRC, KIRP, and LuCa, respectively segregated into the expected cluster based on *SETD2* genotype. It is important to note that this analysis included nonsense, missense, and frameshift mutations, not all of which may impair SETD2 activity and/or H3K36me3 status, and this likely contributes to the “mis-classification” of some tumors. In addition, there are alternative mechanisms by which SETD2 and H3K36me3 can be deregulated, including loss of 3p21, transcriptional regulation, and loss of H3K36me3 substrate (H3K36me1/me2) due to modulation of H3K36me1/me2 histone methyltransferases [36].

**Figure 8:**
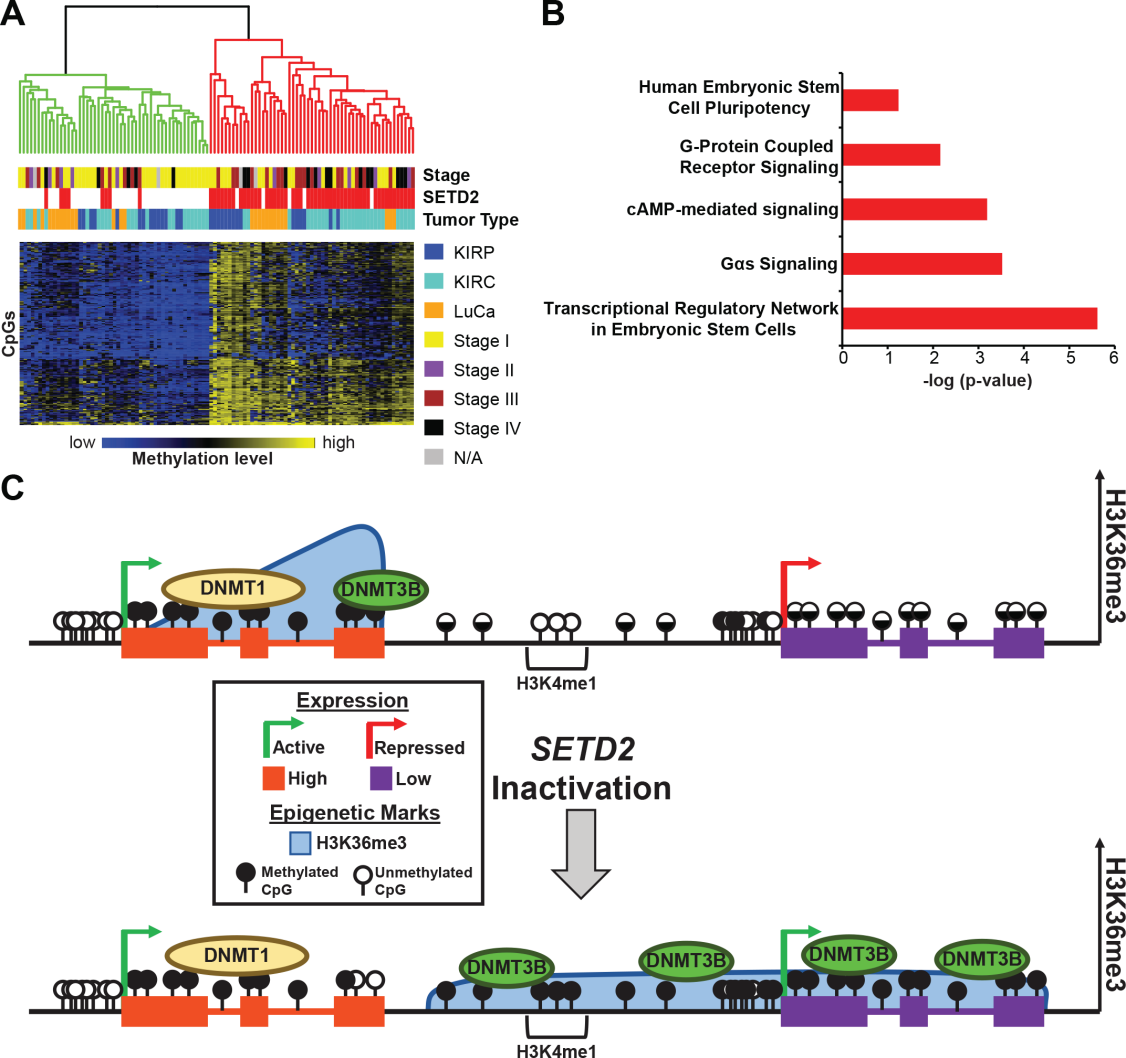
*SETD2* inactivation unveils a hypermethylation signature among tumors induced by dysregulation of epigenetic marks and machinery **A.** Heatmap of the 200 most conserved hypermethylated CpGs in *SETD2* mutant KIRC, KIRP, and LuCa. A color bar is shown with low methylation in blue, intermediate in black, and high methylation in yellow. The two major dendrogram clusters are colored in red and green (*P* = 9.42e-6). Tumor stage classification is provided for each primary tumor. Chi-square testing was used to determine statistical significance between dendrogram clusters and tumor stages (*P* = 2.16e-6). **B.** Ingenuity pathway analysis of the genes associated with the top 1,000 most consistently hypermethylated CpGs in SETD2 mutant tumors (−log p-value). **C.** Model for epigenetic regulation of transcription under conditions of SETD2 inactivation. Top panel: Under normal conditions, high expressing genes (left) are marked with H3K36me3 and 5mC. Low expressing genes (right), have no H3K36me3 and low to intermediate 5mC across the gene body. Intergenic regions also demonstrate intermediate amounts of DNA methylation, and poised enhancers (H3K4me1) lack 5mC. Bottom panel: Under conditions of SETD2 inactivation, H3K36me3 is lost from high expressing genes (left); however, expression remains active and DNMT1 maintains previously established 5mC with the exception of gene termini, as these regions likely maintain their methylation status through the recruitment of DNMT3B. Redistribution of H3K36me3 occurs with loss of SETD2 and spreads across intergenic regions and low expressing genes. DNMT3B is recruited to nascent H3K36me3 through its PWWP domain and establishes new 5mC. Low expressing genes transition to a more transcriptionally active state as a result of deregulated epigenetic patterning.

To better understand the effect of DNA hypermethylation resulting from *SETD2* inactivation in primary tumors, we examined CpGs hypermethylated across all three tumor types (KIRC, KIRP, and LuCa) to determine if there was a common 5mC signature of *SETD2* loss (Figure 8A). Independent of tumor type, a 200 CpG hypermethylation signature was established, demonstrating that loss of SETD2 alters the DNA methylome. Eighty-eight percent of tumors segregated as expected based on *SETD2* genotype. *SETD2* mutant primary tumors derived from KIRC, KIRP, and LuCa analyzed in this study were predominantly associated with high grade (stages III-IV *versus* stages I-II (*p* = 2.16e-6)) and higher stage (*p* = 9.42e-6). We next interrogated the top 1,000 most differentially methylated CpGs between *SETD2* wild-type and mutant tumors using Ingenuity Pathway Analysis (IPA) to better understand the underlying biological processes that may be affected (Figure 8B). The most statistically significant pathway enriched was “Transcriptional Regulatory Network in Embryonic Stem Cells” including genes such as *EOMES, MEIS1,* and *REST*.

To determine if enhancer elements contribute disproportionally to the DNA hypermethylation observed among the three cancer types investigated, as they did in the 786-O cells, we assayed differential 5mC at CpGs within regions marked with histone modifications linked to different types of enhancers (H3K4me1 only, H3K4me1+H3K27ac, H3K27ac only) derived from the Epigenome Roadmap datasets (normal adult kidney was used for KIRC and KIRP; normal adult lung for LuCa). Hypermethylation was consistently enriched among all tumor types at regions marked by K4me1 in normal tissue but was excluded from K27ac-marked regions (Figure S8B). Consistent with our cell line models, SETD2 inactivation induced hypermethylation at K4me1 marked regions across all genomic features (Figure S8C) suggesting that enhancers are a common target for epigenetic deregulation in *SETD2* mutant tumors.

## DISCUSSION

In this study, we used cell culture models and primary tumors to examine how *SETD2* loss-of function mutations drive tumorigenesis. Isogenic 786-O *SETD2* deficient ccRCC cells demonstrated marked redistribution of H3K36me3. While loss of H3K36me3 in gene bodies predominated, substantial ectopic H3K36me3, focused largely on intergenic regions, was also observed. Inactivation of *SETD2* resulted in marked effects on the DNA methylome dominated by genome-wide hypermethylation. DNA hypermethylation significantly co-occurred at sites of ectopic H3K36me3 indicating that this mark profoundly influences 5mC placement. Other regions of the genome without appreciable ectopic H3K36me3 were also subject to DNA hypermethylation, suggesting widespread disruption of 5mC targeting, perhaps due to loss of DNMT3 containment in H3K36me3-rich domains. Redistribution of H3K36 and 5mC resulted in up-regulation of previously non-/low-expressed genes enriched for the poised enhancer mark H3K4me1 in normal adult kidney. Acute depletion of SETD2 in an unrelated *VHL* competent cell line led to a similar effect on 5mC distribution showing that the impact of SETD2 loss-of-function is independent of cell type and method of SETD2 inactivation. Changes in 5mC were conserved in primary ccRCC with biallelic *SETD2* inactivation, in *SETD2*-mutant papillary RCC (a distinct tumor of the kidney), and in lung adenocarcinomas with *SETD2* mutations, and resulted in a distinct 5mC signature that efficiently clustered tumors by *SETD2* genotype and higher tumor grade and stage, consistent with findings by us [2, 13] and others [37-39] that *SETD2* mutations are generally linked to poor prognosis and/or metastasis. Taken together, our results show that *SETD2* mutant tumors represent a new DNA hypermethylator class and that genome-wide redistribution of 5mC caused by SETD2 inactivation, particularly at enhancers, represents one mechanism by which this mutation may promote de-differentiation and cancer progression.

While inactivation of SETD2 in 786-O cells resulted in large-scale losses of H3K36me3, particularly across bodies of high expressing genes that represent the major sink for H3K36me3 in the genome, we unexpectedly also observed ectopic gains in H3K36me3 across low expressed genes and intergenic regions. This finding is consistent, however, with our previous H3K36me3 ChIP-seq analysis of *SETD2* mutant primary ccRCC where we observed ectopic H3K36me3 in a *SETD2* mutant tumor at a region that influenced an RNA splicing event [13]. In the primary tumor analysis it was difficult to rule out intratumoral heterogeneity or normal cell contamination as a cause for ectopic H3K36me3, however our 786-O isogenic model consistently shows overlapping ectopic H3K36me3 peaks with *SETD2* mutant primary ccRCC, underscoring the validity of this finding. SETD2 is thought to be the sole H3K36 trimethylase in mammals [11, 12], although this is largely based on lower sensitivity global quantification methods such as immunohistochemistry or total H3 western blotting. Although we cannot completely rule out the possibility of some residual activity from the SETD2 locus in our 786-O cells, we believe the most likely mediator of the ectopic H3K36me3 is another histone methyltransferase that methylates the H3K36 position, but does not typically perform trimethylation. H3K36me1/me2 are regulated by a diverse group of proteins, including: NSD1 (KMT3B), NSD2 (MMSET/WHSC1), NSD3 (WHSC1L), SETD3, ASHL1, SETMAR (METNASE), and SMYD2. Use of varied substrates, assay conditions, and cell types have likely led to inconsistencies in the reported substrate preferences of each enzyme [40]. The NSD family members, for example, preferentially mono- and dimethylate K36 *in vivo* [40], but are capable of trimethylating K36 *in vitro* [36]. Although we did not observe significant changes in expression of NSD family members in our *SETD2* KO clones (based on RNA-seq, data not shown), it appears plausible one of them could adopt this activity in the absence of normal SETD2 activity and our isogenic 786-O cells represent a good model for identifying this activity. Given that the *SETD2* inactivation-induced ectopic H3K36me3 is linked to genome-wide DNA hypermethylation and gene expression changes associated with dedifferentiation, this activity could represent a novel drug target in *SETD2* mutant tumors.

Prior studies examining the relationship between DNA and H3K36 methylation focused on the impact of H3K36me3 loss to methylated regions of the genome. Hahn *et al*. hypothesized that 5mC and H3K36me3 were established independently since SETD2 depletion did not change 5mC at gene bodies that lost H3K36me3, and conversely H3K36me3 distribution did not change in HCT116 cells depleted of DNMT1 and DNMT3B [41]. Our results showing that highly expressed genes in 786-O *SETD2* KO clones that lost H3K36me3 generally maintained their 5mC supports these observations. The TCGA consortium reported ccRCC DNA hypomethylation was enriched at sites marked by H3K36me3 in normal kidney [3], which is consistent with our findings that 5mC was lost predominantly at H3K36me3-high gene termini. In addition, the TCGA reported DNA hypermethylation focused at CpGs not previously marked by H3K36me3 in normal adult kidney [3]. This also is consistent with our results in that many regions gaining 5mC under SETD2 loss conditions are not marked by H3K36me3, rather it is these loci that gain both ectopic H3K36me3 and 5mC. Finally, our findings are consistent with those of Sato *et al*. who stratified differential 5mC in ccRCCs into three tiers (low, intermediate, and high) and observed that 92% of *SETD2* mutant tumors were present in the intermediate and high 5mC tiers [42]. Thus collectively our findings, supported by the TCGA KIRC dataset, firmly link SETD2 loss-of-function to a global DNA hypermethylation phenotype and more aggressive disease.

Previous work from our laboratory and others showed that DNMT3B was particularly enriched at actively transcribed H3K36me3-marked gene bodies [21, 30, 31], and that H3K36me3 recognition by the DNMT3B PWWP domain is important for its ability to methylate these regions [25, 26]. Based on these studies and our results, we hypothesize that global genome DNA hypermethylation under SETD2 loss-of-function conditions results from two mechanisms (Figure 8C), (*i*) recruitment of DNMT3B to ectopic H3K36me3 regions followed by *de novo* methylation, and (*ii*) loss of normal DNMT3B tethering to gene bodies, allowing it to gain access to normally unmethylated regions of the genome (loss of ‘containment’). We cannot rule out the possible involvement of other DNMTs in this process. In regions already methylated, SETD2 inactivation does not result in 5mC loss because methylation is already established and thus is maintained by DNMT1. The exception to this appears to be gene termini, where loss of H3K36me3 is linked to 5mC loss. Interestingly, ChIP-seq demonstrated DNMT3B was most enriched at gene termini [30], indicating that it might be responsible for both establishment and maintenance of 5mC at gene 3′-ends (Figure 8C). It is therefore of interest to examine whether 5mC regulates aspects of 3′-end processing. Poised normal tissue enhancers were also a prominent target of SETD2 inactivation-induced ectopic H3K36me3 and DNA hypermethylation. Interestingly, active enhancers in human cells are enriched for H3K36me3 [43], consistent with the up-regulation of genes associated with these sequences we observe. The presence of unproductive non-coding RNA transcripts emanating from active enhancers [44] is consistent with acquisition of both H3K36me3 and 5mC, since both marks are recruited to actively transcribed loci. Thus the presence of 5mC at or flanking certain enhancers may be indicative of enhancer activation much in the same way gene body 5mC is linked positively to gene activity [21].

*SETD2* mutation or down-regulation occurs across a broad spectrum of tumor types [3, 35, 38] although in many of these its frequency is relatively low (< 10%) making detailed analysis of its effects feasible only with large datasets. To begin to assess whether the impact of *SETD2* mutations on 5mC localization was conserved in other tumor types, we expanded our analysis to two large public datasets, papillary RCC and lung adenocarcinoma [35]. In both a distinct type of kidney cancer not characterized by chromosome 3p LOH or VHL inactivation, and a tumor of completely different cellular origin, we observed a DNA hypermethylation phenotype strongly linked to *SETD2* mutation. *SETD2* mutations are independently acquired within multiple parts of the same papillary RCC [37], suggesting strong selective pressure to inactivate the K36me3 pathway, and *SETD2* mutations are enriched in relapsed B-ALL [39], reinforcing the link between this mutation and tumor progression. Our results support this notion as a majority of the *SETD2* mutated hypermethylated tumors were associated with more aggressive stage and grade. CIMP (CpG island methylator phenotype) is now recognized in many different tumor types and in the case of glioma is caused by mutations in *IDH1/IDH2*. IDH mutations operate in part by inhibiting TET-mediated DNA demethylation, but also render the tumors more sensitive to DNA hypomethylating agents [45, 46]. Preclincial studies have shown that the DNA methylation inhibitor 5-aza-2′-deoxycytidine (5-azadC) effectively reverses DNA hypermethylation observed in IDH1 mutant gliomas, induces tumor stem cell differentiation, and inhibits tumor growth in mouse models [46]. Our identification of SETD2 as a novel driver of a DNA hypermethylation phenotype suggests that such tumors might also be more susceptible to DNA hypomethylating agents like 5-azadC. Therefore while many chromatin regulator gene mutations are not currently targetable with specific therapies, the interplay between marks, exemplified by 5mC and H3K36me3 described here or IDH1 and 5mC in glioma, suggests that multiple epigenetic regulator mutations may converge on and deregulate 5mC patterns as a common method to promote tumorigenesis. As such, DNA demethylating agents may be more generally applicable as a therapy to target tumors with epigenetic regulator mutations. Our results identify a highly conserved DNA hypermethylation phenotype induced by *SETD2* inactivation that functionally modulates the gene expression program of renal cell cancers, suggesting that DNA demethylating agents represent a potential rational therapy to target *SETD2* loss of function tumors.

## MATERIALS AND METHODS

### Cell culture, SETD2 depletion, DNA/RNA extraction, and quantification of 5mC content by mass spectrometry

786-O parental and *SETD2* KO derivatives were grown in RPMI1640 medium supplemented with 10% heat-inactivated fetal bovine serum and 2 mM L-glutamine. Briefly, *SETD2* was targeted by zinc finger nucleases for deletion and two isogenic clones with frameshifts were generated [13]. SETD2 KO1 contains a 4 base pair deletion and KO2 contains an 11 base pair deletion confirmed by Sanger sequencing [13]. 786-O parental and *SETD2* KO derivatives were validated by cell line authentication provided by ATCC (data available upon request). NCCIT cells were grown in McCoy's 5A medium supplemented with 10% heat-inactivated fetal bovine serum and 2 mM L-glutamine. The On-TARGETplus siRNA SMARTpool (Dharmacon, Thermo Scientific) targeting a single gene was used against *SETD2* (L-012448-00-0005). Transfection with a negative control non-targeting siRNA (D-001206-13-20; Dharmacon, Thermo Scientific) was performed in parallel. SiRNA transfection was performed with PepMute transfection reagent (SignaGen) according to the manufacturer protocol as previously described [31]. Total RNA was extracted by Trizol homogenization and purified according to the manufacturer's protocol (Life Technologies). Genomic DNA was extracted by proteinase K digestion and phenol:chloroform extraction. A portion of this genomic DNA was also used to quantify total genomic 5mC levels by LC-MS/MS exactly as described [32]. Samples for MS were run in duplicate at the Biomarker Mass Spectrometry Facility at the University of North Carolina, Environmental Sciences & Engineering Gillings School of Global Public Health.

### Expression analysis by RNA-seq and qRT-PCR

RNA-seq data was downloaded from the Gene Expression Omnibus (GEO) and aligned to genome build h19 using TopHat v2 [47]. A value of 0.01 was added to all gene RPKM values to account for genes with no expression and prevent artificially large fold-changes in expression [48]. A cutoff of ≥ 2-fold-change in expression was considered differential expression [48]. cDNA synthesis and qRT-PCR was performed in triplicate as described [30]. Primer sequences for ChIP, MeDIP, and qRT-PCR are listed in Table S1 in Supplemental Information.

### Histone extraction and western blotting

Adherent cells were scraped and washed with PBS. Ice-cold lysis buffer (20 mM Tris, 137 mM NaCl, 1.5 mM MgCl_2_, 1mM EDTA, 10% Glycerol, 1% Triton X-100) with protease inhibitors was added to the cell pellet (900 μl/100 mm dish). The lysis reaction was sonicated on ice at 40W for 30 seconds at 50% duty (Branson 450 Sonifier) and centrifuged at 13,200 RPM for 20 minutes at 4°C. The supernatant was collected and assayed for protein concentration using BCA. Protein extracts (2 μg) were size-separated on an 18% acrylamide gel and transferred to PVDF membrane. Membranes were blocked for one hour at room temperature using 5% BSA/0.1% TBST (50 mM Tris-HCl, pH 7.5, 150 mM NaCl, 0.1% Tween 20). Membranes were then incubated with the primary antibody for 1.5 hours at room temperature and rinsed four times in 0.1% TBST. Membranes were incubated in appropriate secondary antibody diluted 1:20,000 in 5% BSA/0.1% TBST for one hour at room temperature and rinsed. Pierce Pico was added for five minutes at room temperature for detection. Antibodies and dilutions used are: histone H3 (Abcam 1791 1:1000), H3K36me1 (Abcam 9048 1:1000), H3K36me2 (Abcam 9049 1:1000), H3K36me3 (Abcam 9050 1:1000). Dot blotting was performed following the Abcam protocol.

### MeDIP pull-down assays

MeDIP experiments were performed as previously described [31] with the 33D3 5mC antibody (Diagenode).

### ChIP-qPCR

ChIP-pull downs for H3K36me3 (Active Motif 61021) were performed using an in-house protocol as previously described [13] and detailed in Supplemental Information.

### ChIP-seq data analysis

ChIP-seq data processing was conducted as previously described [30] and detailed in Supplemental Information.

### 450K array data analysis

DNA samples were processed on the HumanMethylation450 BeadChip array (Illumina) and analyzed as previously described [31, 49] and detailed in the Supplemental Information.

### Gene ontology and pathway analysis

Ontology analysis was performed using GO_BP within the DAVID bioinformatics database with Benjamini correction for multiple testing [50] or Ingenuity Pathway Analysis (Qiagen) using standard program parameters.

### Significance testing

The Fisher Exact test with a two-tailed *p*-value calculation was used for testing the significance of data set comparisons as described previously for similar data sets [51]. For added stringency, a modified EASE score was applied to all Fisher Exact tests. Chi-square testing was used to determine significance of clustering and tumor grade.

### TCGA sample IDs

Gene expression, exome sequencing, 450K array, and tumor grade data was generated by the Cancer Genome Atlas and downloaded from http://cancergenome.nih.gov/. Patient sample identification numbers used for KIRC, KIRP, and LuCa 450K array analysis are provided in Tables S2, S3, and S4, respectively in Supplemental Information. Data was downloaded from TCGA on 4/23/2015.

### Availability of supporting data

450K array data for 786-O parental, *SETD2* KO1 and KO2, and siKD of SETD2 in NCCIT cells have been deposited in GEO (GSE70645). NCCIT no-target control (NTC) was previously deposited to GEO under accession GSE54840 (sample GSM1527531).

Previously released dataset accession numbers are provided in Table S5 in Supplemental Information.

## SUPPLEMENTARY MATERIAL FIGURES AND TABLES


