# Supplementary material for: Dynamic reprogramming of DNA methylation in SETD2-deregulated renal cell carcinoma
Source: Oncotarget. 2015 Dec 5;7(2):1927–46. doi: 10.18632/oncotarget.6481 (PMC4811507; doi:10.18632/oncotarget.6481)
Supplement: Supplementary file 1 [file oncotarget-07-1927-s001.pdf]

Dynamic reprogramming of DNA methylation in SETD2-deregulated renal cell carcinoma

Supplementary Material

A

Genotype and ZFN Information:

EXON SEQUENCE = all caps
Intron sequence = lowercase
Primer Binding Site = bold & underlined
ZFN binding = bold & red
ZFN CUT SITE = red & underlined
DELETED NT REPLACED BY -----

786-O Parental *SETD2* WT

CC**ACTTTCCAAAACAGGCCAGA**TAGTAGACTGGGAAAAACAGAATTGAGTTTTTCTTCCTCTTGTGAGATACCACA
TGTGGATGGCTTGCACTCATCAGAAGAGCTCAGAAACTTAGGTTGGGACTTCTCTCAAGAAAAGCC**TTCTACCACG**
TATCAGCAACCTGACAGTAGCTATGGAGCTTGTGGTGGACACAAGTATCAGCAAAATGCAGAACAGTATGGTGGGA
CACGTGATTACTGGCAAGGCAATGGTTACTGGGATCCAAGATCAGGTAGACCTCCTGGAACTGGGGTTGTGTATGA
TCGAACTCAAGGACAAGTACCAGATTCCCTAACAGATGATCGTGAAGAAGAGGAGA**ATTGGGATCAACAGGATGGA**

786-O *SETD2* KO1

CC**ACTTTCCAAAACAGGCCAGA**TAGTAGACTGGGAAAAACAGAATTGAGTTTTTCTTCCTCTTGTGAGATACCACA
TGTGGATGGCTTGCACTCATCAGAAGAGCTCAGAAACTTAGGTTGGGACTTCTCTCAAGAAAAGCC**TTCTACCACG**
TATCAGCAACCTGACAGTA----TGGAGCTTGTGGTGGACACAAGTATCAGCAAAATGCAGAACAGTATGGTGGGA
CACGTGATTACTGGCAAGGCAATGGTTACTGGGATCCAAGATCAGGTAGACCTCCTGGAACTGGGGTTGTGTATGA
TCGAACTCAAGGACAAGTACCAGATTCCCTAACAGATGATCGTGAAGAAGAGGAGA**ATTGGGATCAACAGGATGGA**

786-O *SETD2* KO2

CC**ACTTTCCAAAACAGGCCAGA**TAGTAGACTGGGAAAAACAGAATTGAGTTTTTCTTCCTCTTGTGAGATACCACA
TGTGGATGGCTTGCACTCATCAGAAGAGCTCAGAAACTTAGGTTGGGACTTCTCTCAAGAAAAGCC**TTCTACCACG**
TATCAGCAACCTGACAG-----------CTTGTGGTGGACACAAGTATCAGCAAAATGCAGAACAGTATGGTGGGA
CACGTGATTACTGGCAAGGCAATGGTTACTGGGATCCAAGATCAGGTAGACCTCCTGGAACTGGGGTTGTGTATGA
TCGAACTCAAGGACAAGTACCAGATTCCCTAACAGATGATCGTGAAGAAGAGGAGA**ATTGGGATCAACAGGATGGA**

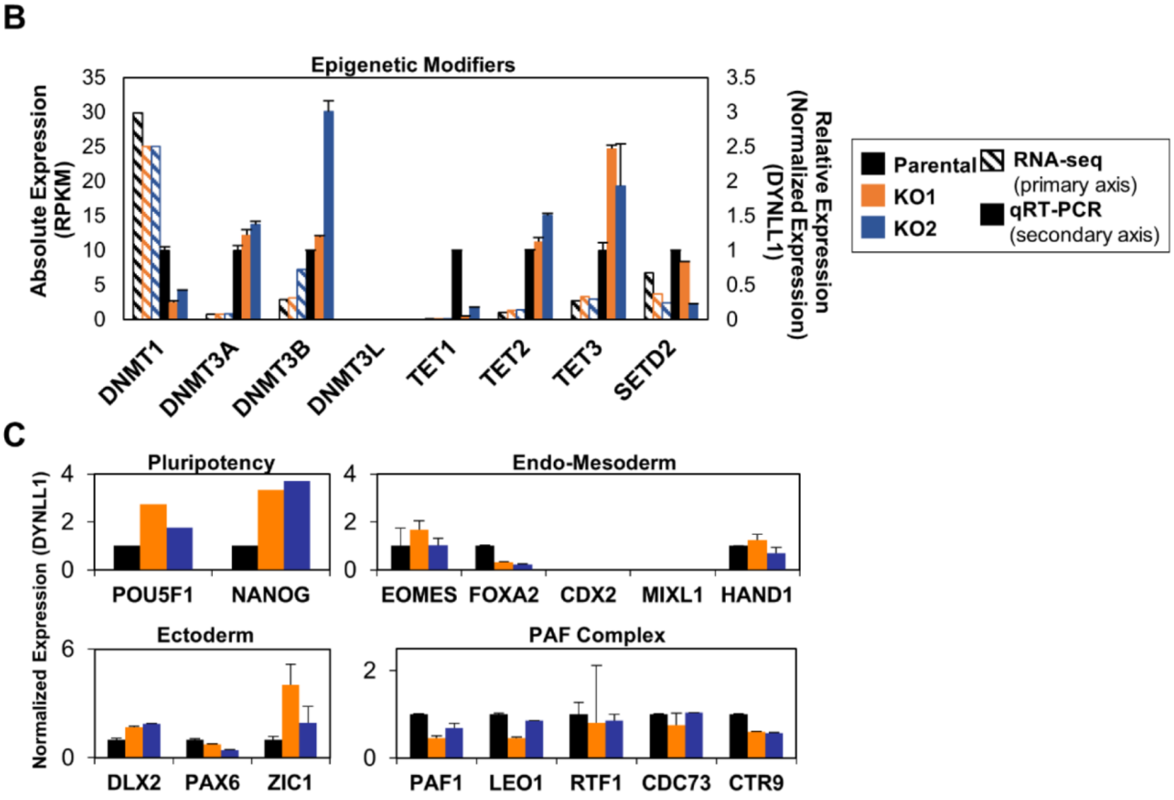

Figure S1. Validation of ccRCC cell line models, related to Figure 1. (A) Schematic representations of the ZFN targeting strategy of SETD2, and the effective deletion of 4 bp in KO1 and 11 bp in KO2 clones. **(B)** Absolute (RNA-seq) and relative (qRT-PCR) mRNA expression of epigenetic modifiers. RNA-seq is plotted on the left axis and qRT-PCR data is plotted on the right axis. For qRT-PCR, error bars represent standard error of triplicate reactions. **(C)** Relative mRNA expression measured by qRT-PCR of genes involved in transcription and developmental pathways.

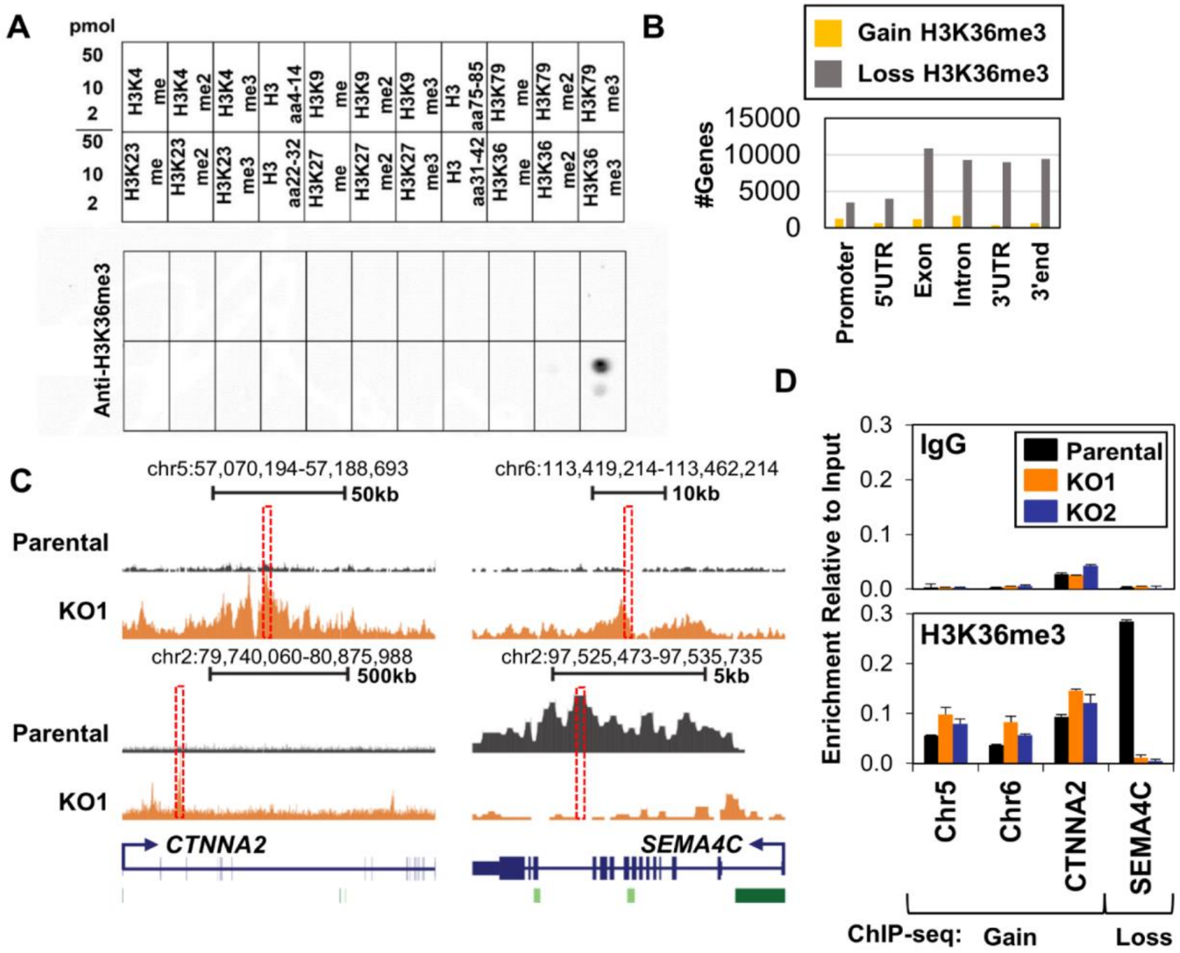

Figure S2. Validation of H3K36me3 ChIP-seq, related to Figure 1. (A) Dot blot demonstrating specificity of Active Motif H3K36me3 antibody for H3K36me3 histone mark. **(B)** Number of genes with ≥ 2-fold-change in H3K36me3 level for SETD2 KO clone 1. **(C)** Representative browser shots demonstrating loss and gain of H3K36me3 in SETD2 KO clone 1. Top panel represents intergenic regions that gain H3K36me3. Dotted red boxes indicate relative position of regions assayed by ChIP-qPCR in SF2D. Bent arrows = TSS, Green bars = CpG islands. **(D)** H3K36me3 ChIP-qPCR of candidate regions for gain and loss of H3K36me3. IgG serves as negative control for ChIP.

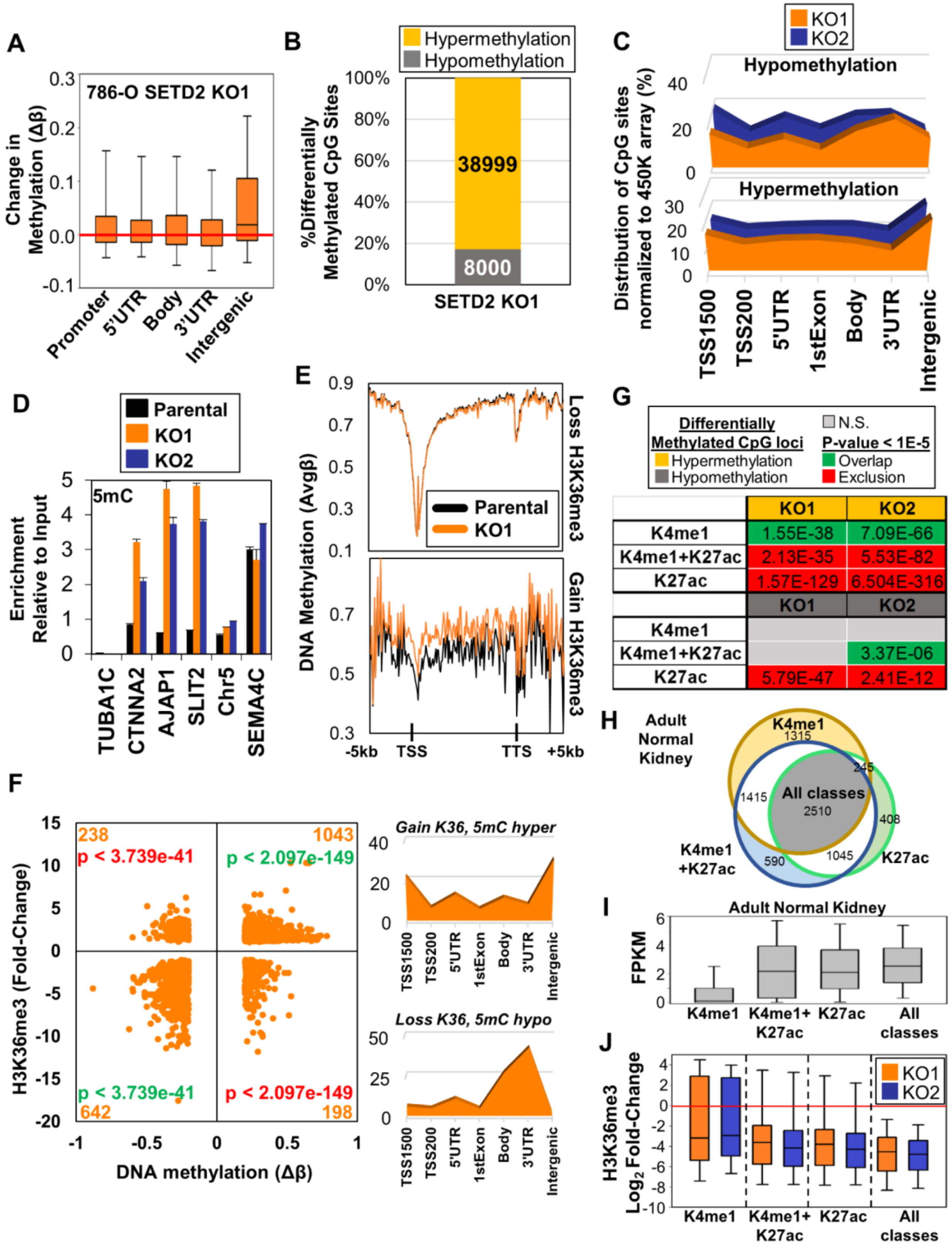
A
786-O SETD2 KO1
Change in Methylation (Δβ)
0.3
0.2
0.1
0.0
-0.1
Promoter
5'UTR
Body
3'UTR
Intergenic
B
Hypermethylation
Hypomethylation
%Differentially Methylated CpG Sites
100%
80%
60%
40%
20%
0%
38999
8000
SETD2 KO1
C
KO1
KO2
Hypomethylation
Hypermethylation
Distribution of CpG sites normalized to 450K array (%)
40
20
0
30
20
10
0
TSS1500
TSS200
5'UTR
1stExon
Body
3'UTR
Intergenic
D
Parental
KO1
KO2
5mC
Enrichment Relative to Input
5
4
3
2
1
0
TUBA1C
CTNNA2
AJAP1
SLIT2
Chr5
SEMA4C
E
DNA Methylation (Avgβ)
0.9
0.7
0.5
0.3
0.1
0.7
0.5
0.3
Parental
KO1
Loss H3K36me3
Gain H3K36me3
-5kb
TSS
TTS
+5kb
G
Differentially Methylated CpG loci
Hypermethylation
Hypomethylation
N.S.
P-value < 1E-5
Overlap
Exclusion
	KO1	KO2
K4me1	1.55E-38	7.09E-66
K4me1+K27ac	2.13E-35	5.53E-82
K27ac	1.57E-129	6.504E-316
	KO1	KO2
K4me1		
K4me1+K27ac		3.37E-06
K27ac	5.79E-47	2.41E-12
H
Adult Normal Kidney
K4me1
1315
245
1415
All classes
2510
408
590
1045
K4me1 +K27ac
K27ac
F
H3K36me3 (Fold-Change)
15
10
5
0
-5
-10
-15
-20
238
p < 3.739e-41
1043
p < 2.097e-149
p < 3.739e-41
642
p < 2.097e-149
198
-1
-0.5
0
0.5
1
DNA methylation (Δβ)
Gain K36, 5mC hyper
40
20
0
Loss K36, 5mC hypo
50
25
0
TSS1500
TSS200
5'UTR
1stExon
Body
3'UTR
Intergenic
I
Adult Normal Kidney
FPKM
6
4
2
0
K4me1
K4me1+ K27ac
K27ac
All classes
J
H3K36me3 Log2 Fold-Change
4
2
0
-2
-4
-6
-8
-10
KO1
KO2
K4me1
K4me1+ K27ac
K27ac
All classes

Figure S3. Distribution of DNA methylation genome-wide, integration with H3K36me3, and confirmation of 450K array data in SETD2 KO1 clone, related to Figure 2. (A) Box plots representing all Δβ-values (change in 5mC) in the indicated features. **(B)** Percentage of differentially methylated CpG sites (lΔβl ≥ 0.2) classified by methylation change upon SETD2 KO (hypo/hypermethylation). **(C)** Normalized distribution of significantly differentially methylated CpG sites by genomic feature. **(D)** MeDIP analysis of candidate hypermethylated regions in 786-O SETD2 KO cells using the 33D3 (Diagenode) 5mC antibody to pull-down methylated DNA followed by qPCR to analyze relative enrichment at the indicated genes. *TUBA1C* serves as a negative control for all samples. *SEMA4C* serves as a positive control region for DNA methylation that does not change with SETD2 KO. **(E)** Spatial distribution plots of DNA methylation across intragenic regions derived from average β-values stratified by loss or gain in H3K36me3 upon SETD2 KO in clone 1. **(F)** Left: Scatter plot of differentially methylated CpGs (lΔβl ≥ 0.2) within peaks of H3K36me3 in 786-O cells indicating the change in methylation (x-axis) versus change in H3K36me3 (y-axis) in clone 1. Number of CpGs is provided in the corner of each quadrant along with significance of the methylation/H3K36me3 overlap. Green = significant overlap; Red = significant exclusion. Right: Genomic feature enrichment plots, normalized to the number of CpGs present in each feature on the 450K array, for the categories with significant overlap. **(G)** Overlap analysis of SETD2 KO-induced differentially methylated CpGs in regions with the indicated enhancer marks (Fisher's Exact test). N.S. = Not significant **(H)** Venn diagram showing overlap of genes that contain regions marked by K4me1, K27ac, or both K4me1 and K27ac in human normal adult kidney. *Color regions indicate genes that are used in Figures 2F and SF3I-J. **(I)** Box plots of mRNA expression (from RNA-seq) for genes classified by exclusive regions of particular enhancer-mark status (K4me1 only, K27ac only, K4me1+K27ac only) from normal adult kidney. "All classes" indicates loci with all enhancer

groups. **(J)** Box plots of H3K36me3 change in 786-O SETD2 KO cells for genes marked by the specified enhancer marks in normal adult kidney (see SF3H).

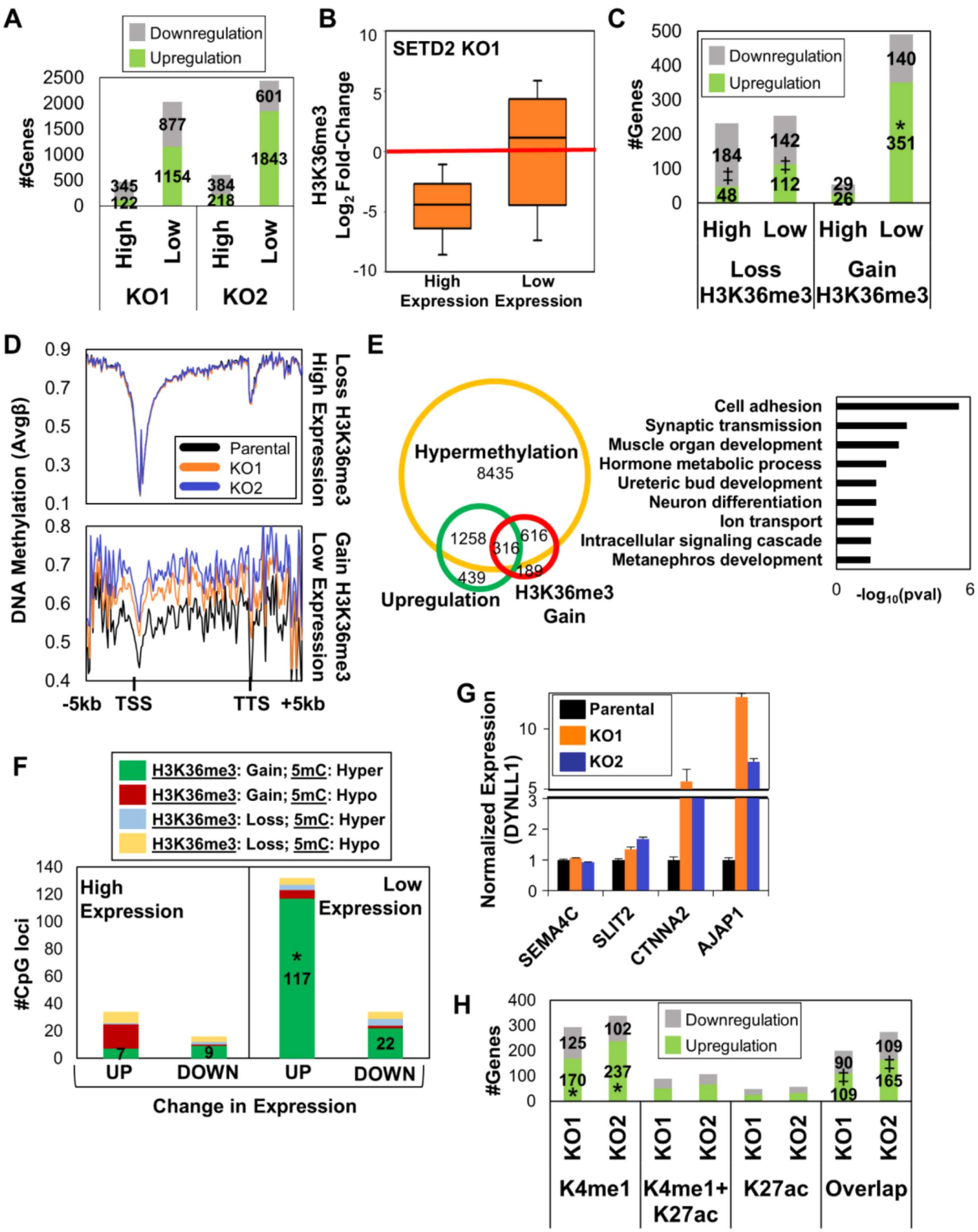

A
Downregulation
Upregulation
#Genes
2500
2000
1500
1000
500
0
345
122
877
1154
384
218
601
1843
High
Low
High
Low
KO1
KO2
B
SETD2 KO1
H3K36me3 Log2 Fold-Change
10
5
0
-5
-10
High Expression
Low Expression
C
#Genes
500
400
300
200
100
0
Downregulation
Upregulation
184
‡
48
142
‡
112
29
26
140
*
351
High
Low
High
Low
Loss H3K36me3
Gain H3K36me3
D
DNA Methylation (Avgβ)
0.9
0.7
0.5
0.3
0.1
Parental
KO1
KO2
Loss H3K36me3 High Expression
0.8
0.7
0.6
0.5
0.4
Gain H3K36me3 Low Expression
-5kb
TSS
TTS
+5kb
E
Hypermethylation
8435
1258
316
616
439
189
Upregulation
H3K36me3 Gain
Cell adhesion
Synaptic transmission
Muscle organ development
Hormone metabolic process
Ureteric bud development
Neuron differentiation
Ion transport
Intracellular signaling cascade
Metanephros development
0
-log10(pval)
6
F
H3K36me3: Gain; 5mC: Hyper
H3K36me3: Gain; 5mC: Hypo
H3K36me3: Loss; 5mC: Hyper
H3K36me3: Loss; 5mC: Hypo
#CpG loci
140
120
100
80
60
40
20
0
High Expression
Low Expression
7
9
*
117
22
UP
DOWN
UP
DOWN
Change in Expression
G
Normalized Expression (DYNLL1)
Parental
KO1
KO2
10
5
3
2
1
0
SEMA4C
SLIT2
CTNNA2
AJAP1
H
#Genes
400
300
200
100
0
Downregulation
Upregulation
125
170
*
102
237
*
90
‡
109
109
‡
165
KO1
KO2
KO1
KO2
KO1
KO2
KO1
KO2
K4me1
K4me1+ K27ac
K27ac
Overlap

Figure S4. Redistribution of H3K36me3 and DNA methylation, and their links to altered gene expression with SETD2 KO in clone 1, related to Figure 3. (A) Number of genes up-/down-regulated (≥ 2 fold-change) at high and low expressing genes in parental 786-O cells. **(B)** Box plot showing the fold-change in H3K36me3 relative to parental 786-O cells at high and low expressing genes. RPKM values stratified expression into high and low tiers. **(C)** Number of genes up-/down-regulated (≥ 2 fold-change) in each expression tier (high/low) classified by whether the gene demonstrated loss or gain in H3K36me3 upon SETD2 KO. *=overlap (pval<E-50), ‡=exclusion (pval<E-50) **(D)** Spatial distribution plots of 5mC across intragenic regions derived from average β-values stratified by loss or gain in H3K36me3 and expression tier (high/low). **(E)** Left. Overlap of genes that are hypermethylated ($\Delta\beta \geq 0.2$), up-regulated (≥ 1.5 fold-change), and gain H3K36me3 (≥ 2 fold-change) with SETD2 KO in clone 2. Right. Ontology for genes that overlap all three categories. **(F)** Number of CpGs that demonstrate the indicated changes in H3K36me3 and 5mC stratified by expression tier and expression change. *=overlap (pval<E-10). **(G)** Relative mRNA expression measured by qRT-PCR of candidate genes that gain H3K36me3 and 5mC, and become up-regulated based on RNA-seq. Error bars represent standard error of triplicate reactions. **(H)** Number of genes up-/down-regulated with SETD2 KO stratified by enhancer classification status from SF3H. *=overlap (pval<E-10), ‡=exclusion (pval<E-5)

A

	KO1		KO2		Conserved	
	#DMRs	#CpGs	#DMRs	#CpGs	#DMRs	#CpGs
**Total**	580	7151	1928	26069	467	5770
**TSS1500**	78	903	293	3383	52	678
**TSS200**	41	718	139	2358	34	557
**5'UTR**	50	666	157	1932	35	480
**1stExon**	31	345	99	1146	23	259
**Body**	160	1895	509	7291	133	1593
**3'UTR**	10	131	54	532	10	113
**Intergenic**	210	2493	677	9427	180	2090

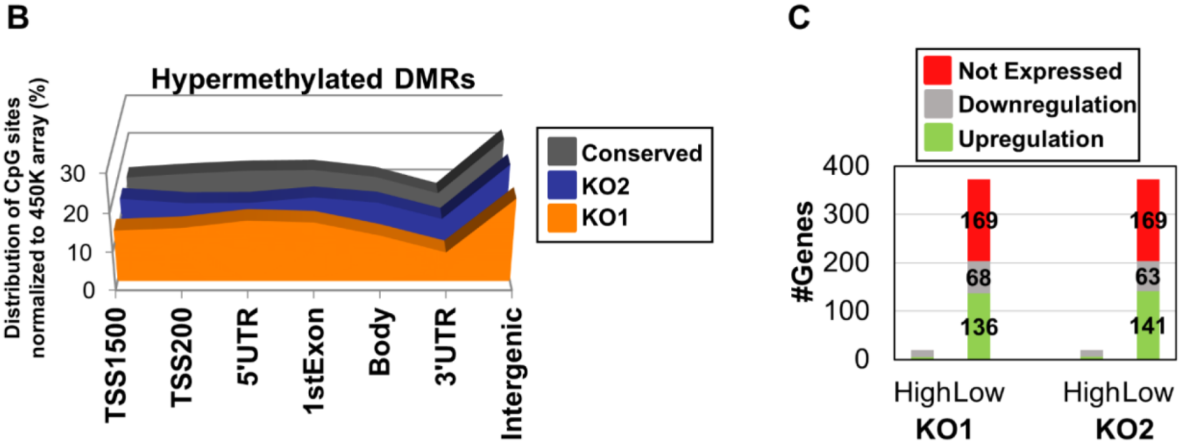

Figure S5. Conservation of hypermethylated DMRs between independent SETD2 KO clones, related to Figure 4. (A) Table indicating the number of DMRs (≥ 8 CpG loci with $\Delta\beta \geq 0.20$) and CpGs contributing to DMRs for each SETD2 KO clone and the number of DMRs and CpGs conserved between the two independent SETD2 KO clones. **(B)** Normalized distribution of significantly hypermethylated CpGs within DMRs by genomic feature. **(C)** Number of genes with indicated gene expression changes for genes within hypermethylated DMRs stratified by expression tier.

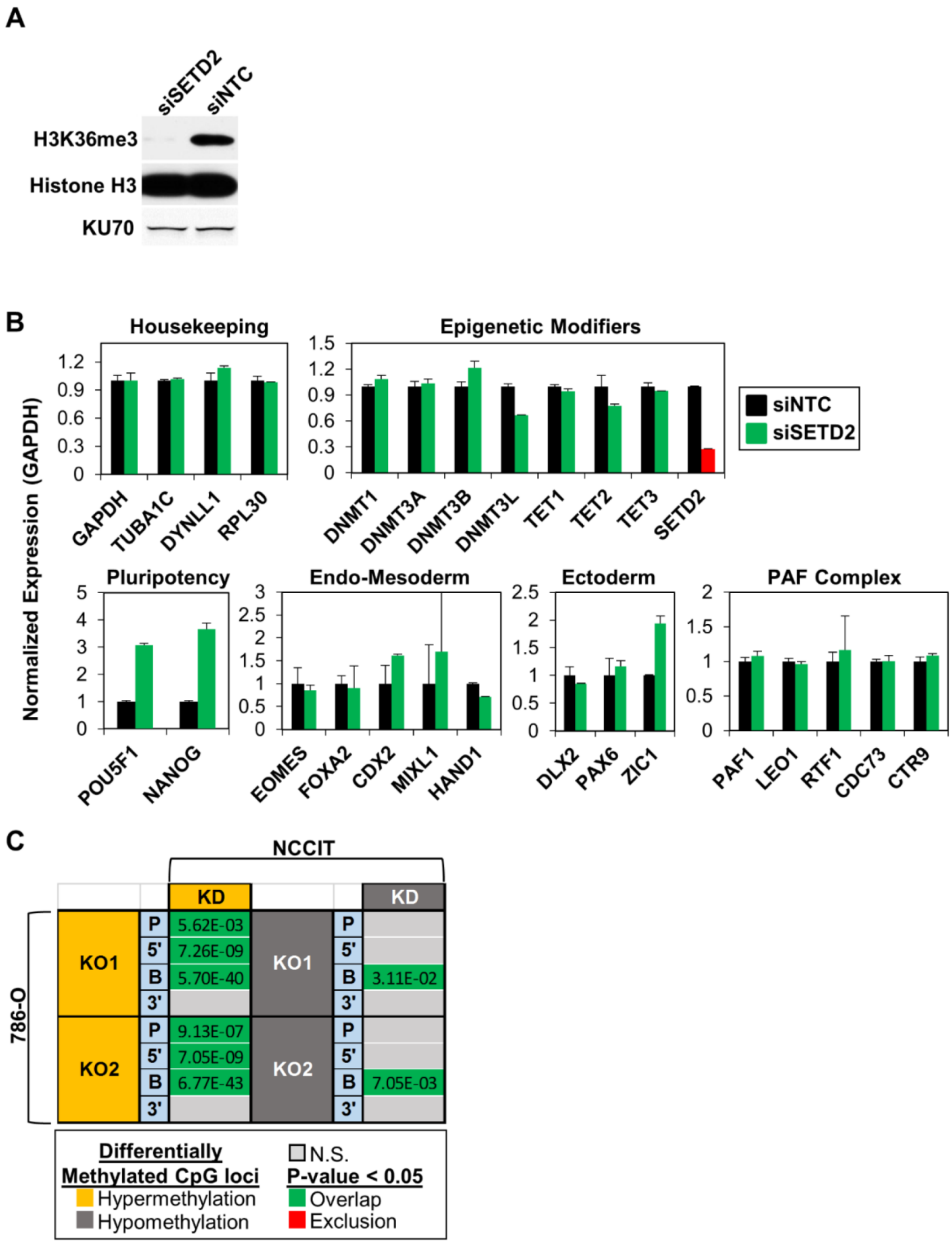

Figure S6. Validation of SETD2 siRNA knockdown in NCCIT cells and conservation of methylation changes with the 786-O SETD2 KO model, related to Figure 5. (A) Western

blot for H3K36me3 in NCCIT SETD2 siKD cells. Histone H3 and KU70 serve as loading controls. **(B)** Relative mRNA expression measured by qRT-PCR of genes involved in housekeeping, epigenetic, transcription, developmental pathways, and the SETD2 siKD level. Error bars represent standard error of triplicate reactions. **(C)** Overlap analysis of genes that contain differentially methylated CpGs (SETD2 KD = $|\Delta\beta| \geq 0.1$; SETD2 KO = $|\Delta\beta| \geq 0.2$). The Fisher's Exact Test was used to determine significance.

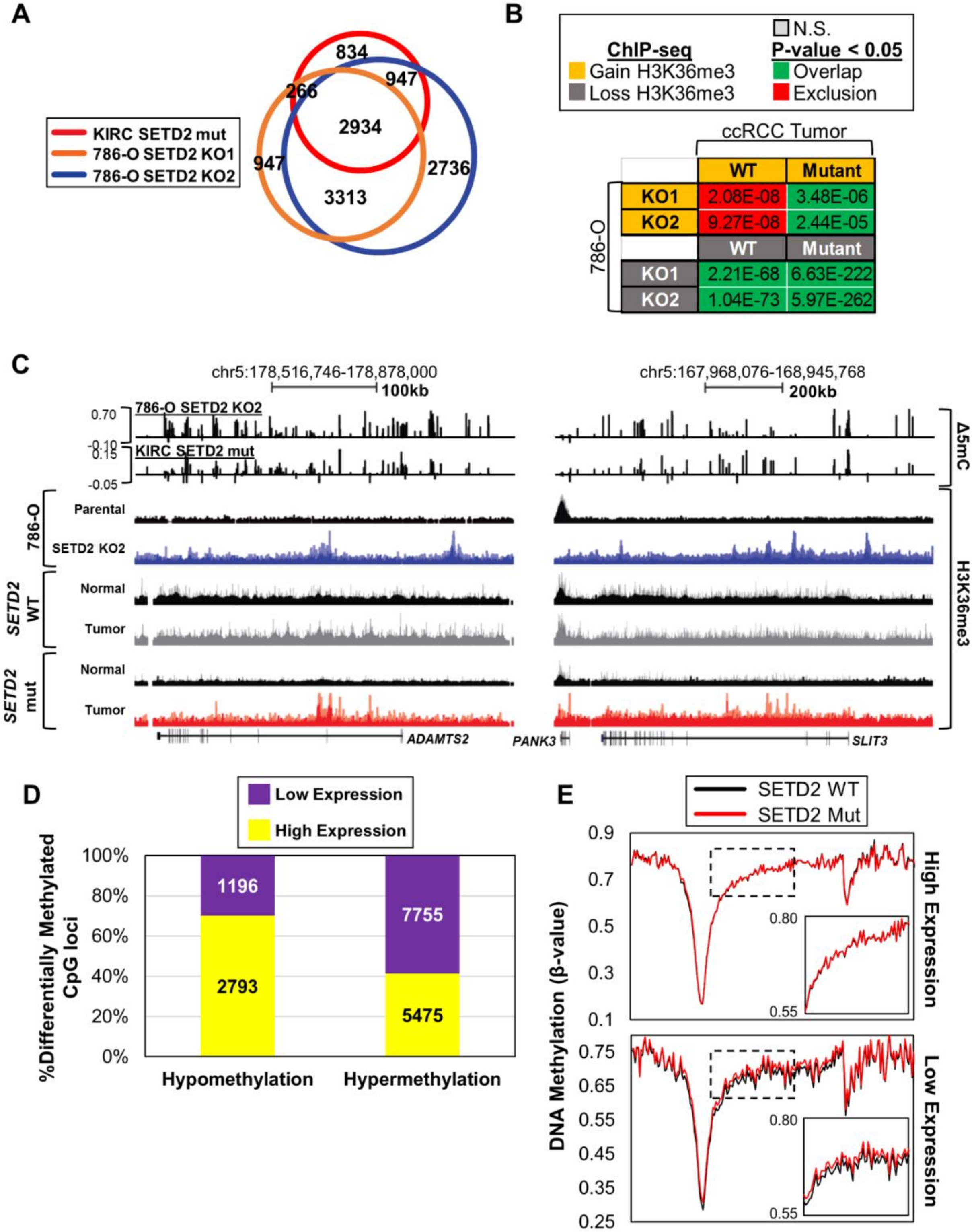

A
834
947
266
2934
947
2736
3313
KIRC SETD2 mut
786-O SETD2 KO1
786-O SETD2 KO2
B
N.S.
ChIP-seq
P-value < 0.05
Gain H3K36me3
Loss H3K36me3
Overlap
Exclusion
ccRCC Tumor
786-O
WT
Mutant
KO1 2.08E-08 3.48E-06
KO2 9.27E-08 2.44E-05
KO1 2.21E-68 6.63E-222
KO2 1.04E-73 5.97E-262
C
chr5:178,516,746-178,878,000
100kb
chr5:167,968,076-168,945,768
200kb
786-O SETD2 KO2
KIRC SETD2 mut
Δ5mC
Parental
SETD2 KO2
Normal
Tumor
SETD2 WT
SETD2 mut
H3K36me3
ADAMTS2
PANK3
SLIT3
D
Low Expression
High Expression
%Differentially Methylated CpG loci
1196
2793
7755
5475
Hypomethylation
Hypermethylation
E
SETD2 WT
SETD2 Mut
DNA Methylation (β-value)
High Expression
Low Expression

Figure S7. Epigenetic changes in SETD2 inactivation cell line models are recapitulated in *SETD2* mutated primary ccRCC, related to Figure 6. (A) Venn diagram summarizing overlap of hypermethylated genes in 786-O SETD2 KO cells and ccRCC *SETD2* mutated tumors. Ontology analysis is presented in Figure 6F. **(B)** Overlap analysis of genes that gain/lose H3K36me3 in 786-O SETD2 KO cells and two primary ccRCCs that harbor WT *SETD2* or mutant *SETD2* (biallelic inactivation), respectively. Fisher's Exact Test was used to determine significance. **(C)** Browser shots of regions that gain H3K36me3 in the 786-SETD2 KO2 clone (relative to parental) and the primary *SETD2* mutated ccRCC tumor (relative to matched adjacent normal kidney tissue), but do not show altered H3K36me3 in the *SETD2* WT primary ccRCC tumor. The TCGA KIRC dataset is presented for 5mC analysis. **(D)** Percentage of differentially methylated CpGs ($|\Delta\beta| \geq 0.1$) within the respective expression level tiers. **(E)** Spatial distribution plots of DNA methylation in ccRCC tumors stratified by expression level of genes in the matched normal adjacent kidney patient samples. Dotted box indicates region that is enlarged in bottom right of plot to better visualize the change in methylation that occurs at lower expression genes.

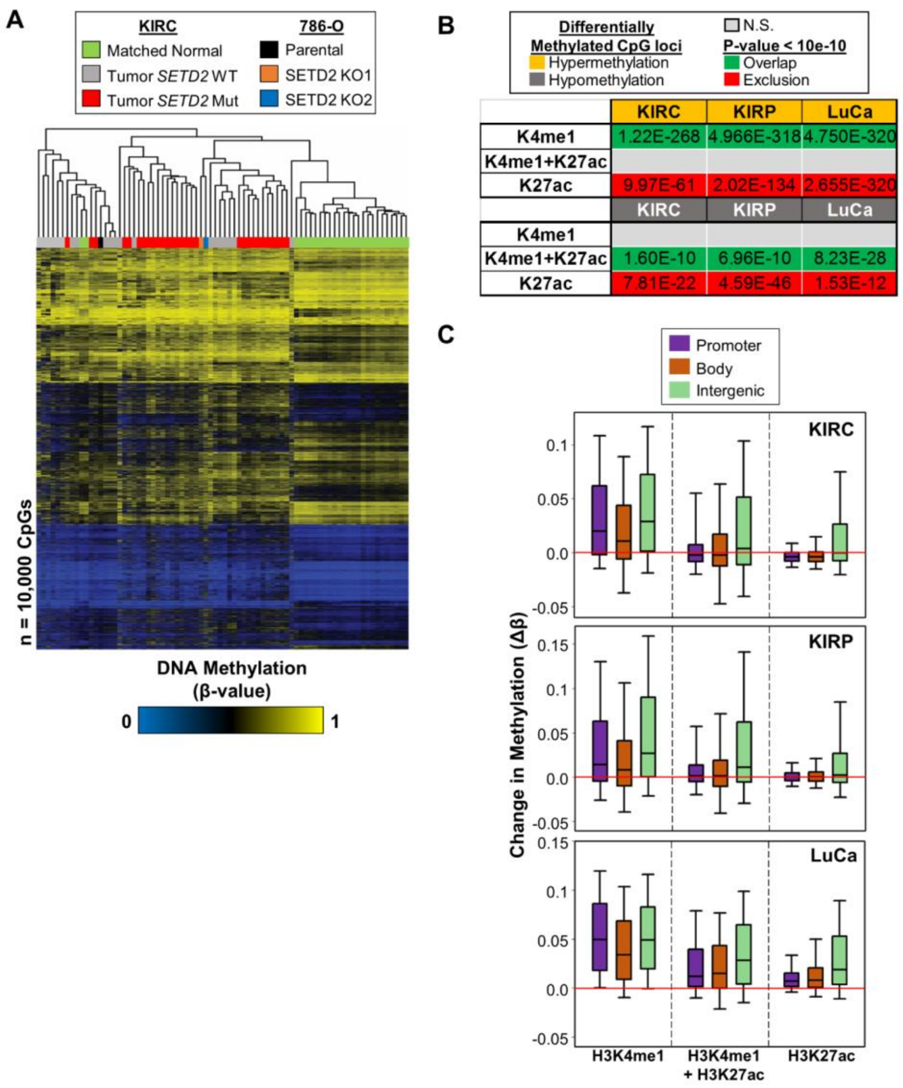

Figure S8. Mutation of *SETD2* in tumors induces DNA hypermethylation at poised enhancers, related to Figure 8. (A) Unsupervised clustering of the top 10,000 most differentially methylated CpGs (calculated by standard deviation) among primary ccRCCs (KIRC) and 786-O cell line samples. **(B)** Overlap analysis of differentially methylated CpGs (|Δβ| ≥ 0.1) in *SETD2* mutated tumors within peaks of the indicated enhancer mark(s). Fisher's Exact Test was used to determine level of significance. **(C)** Box plots representing all Δβ-values (change in methylation) for CpGs within peaks of the respective enhancer mark(s) for each tumor type.

Table S1. Primer sequences for qRT, MeDIP, ChIP-PCR. *indicates primer sequences from Ehrlich *et al*. [1]

**QRT-PCR**	**Forward primer**	**Reverse primer**
GAPDH	CTTTGGTATCGTGGAAGGACTC	GTAGAGGCAGGGATGATGTTC
DNMT1	GGAGAGGCTAAGCGTTCAAG	AAATGAGATGTGATGGTGGTTTG
DNMT3A	AAGAGCACAGCGGAGAAG	GCAGATGTCCTCAATGTTCC
DNMT3B*	CCATGAAGGTTGGCGACAA	TGGCATCAATCATCACTGGATT
DNMT3L*	GGGACAACTGAAGCATGTGGT	AAGATCGAAGGGTCCCCACT
TUBA1C	CCGGGCAGTGTTTGTAGACTTGG	ATCTCCTTGCCAATGGTGTAGTGCC
DYNLL	CATAGAGAAGGACATTGCGGCTCATATC	GAACAGAAGAATGGCCACTTGGC
RPL30	TTGAACTGGGCACAGCATGCGG	CTTTTCACCAGTCTGTTCTGGCATGC
OCT4	CTTGCTGCAGAAGTGGGTGGAGGAA	CTGCAGTGTGGGTTTCGGGCA
NANOG	GAACTCTCCAACATCCTGAACC	TTCTGCGTCACACCATTGC
HAND1	AAAGGCTCAGGACCCAAGAA	CAGCACGTCCATCAGGTAGG
CDX2	GCTACATCACCATCCGGAG	GCTGCTGCAACTTCTTCTTG
DLX2	AGACTCAATACTTGGCCTTGC	GCGAAGCACAAGGTGGAGAAGCG
ZIC1	CAAGTCCTACACGCATCCCAG	CGTGGAGGATTCGTAGCCAGAG
FOXA2	TATGCTGGGAGCGGTGAAGATGG	CGTGTTCATGCCGTTCATCC
MIXL1	CAAGCGCACGTCTTTCAGC	GCACAGTGGTTGAGGATAATCT
PAX6	GCCCGGCCGTGCGACATTTCC	GCACTTGGACTTTTGCATCTGCATGGG
EOMES	GGCAAATGGGTGACCTGTG	GAAATCTCCTGTCTCATCC
TET1	GGGCACCCTACCGACAGAAGATGC	CTTCTGGGGCTTGGGCTTCTACC
TET2	GGATGTCCTATTGCTAAGTGG	GAATCACAATCACTGCAGCCTC
TET3	GAGCTGGCGGGCATTACG	TGCGGCTCCACCTTGAGG
SETD2	TGGACTGTGAACGGACAACTGAGG	GGCAATTGGCTGATCCGCAGAAAC
LEO1	CAATGGCTGGTCGTGATCCTGA	GCGCTGCTGAGATTCCCTACGT
RTF1	GCGGATTGGCATCGGAAACCACA	AGCGTTCTAGCTTATGCCGTGAT
CDC73	CCTTATAGAGTAGTAGACCAGCCCC	CCATGGCCAACCTTTGAACTG
CTR9	CCCTTTCAGCCTATGGAACAGCAA	CTTTTGCACGGTCCAATGACGC
SEMA4C	ATGTACCTGAGAGTGTGGGCA	ATATCGCCCTTGCAGACACG
SLIT2	CTTGGTTGGGAGAGTGGCTGA	CAGTAGGACAGCGAGAAAGTGGG
CTNNA2	TGGAGCCACTTGTTACACAG	GCTCTACAGAGGCAGCTAGTACAT
AJAP1	CATCACCATCACCGTCTCCCTCAT	AAGTCCGTGAGGTTCTGGCAG
**MeDIP**	**Forward primer**	**Reverse primer**
TUBA1C	TCACTACTTCTCCCCCGGACT	CCCACTCACCATGACTAGAACTTG
CTNNA2	TGGAGCCACTTGTTACACAG	GCTCTACAGAGGCAGCTAGTACAT
AJAP1	TTAGGAGGAAGCCCGTCTTGTGG	TCATGAACTCGCCGGGGATCCTCT

SLIT2	GATAGGGGACAAGTACTGGAGGA	GAGGACTGGGCTCTGAAACC
Chr5	CTGTTGGCTGGTGCAATGAG	ACAAGACCATAAGCCGTGGA
SEMA4C	TCGGCATAGCAGTCGGACTCCA	CCTGAGAGTGTGGGCAGCTTCA
**ChIP**	**Forward primer**	**Reverse primer**
Chr5	CTGTTGGCTGGTGCAATGAG	ACAAGACCATAAGCCGTGGA
Chr6	TTGGCAAAATTTCCTGCCGTAT	AACACCAAGCAACCTTAAAGCC
CTNNA2	GGGTATATTGCTTCCAGGTAGTGAGC	GGGGAGCTAAACACTAAGCACATAT
SEMA4C	TCGGCATAGCAGTCGGACTCCA	CCTGAGAGTGTGGGCAGCTTCA

Table S2. TCGA samples used from KIRC dataset

TCGA ID	SETD2 status	450K chip and location
TCGA-CJ-4912	WT	6042308129_R01C02
TCGA-B0-5400	WT	6042308157_R04C01
TCGA-BP-4770	WT	6042308163_R06C02
TCGA-B0-5080	WT	6042316017_R01C02
TCGA-B8-4621	WT	6042316017_R02C01
TCGA-B0-5100	WT	6042324027_R01C01
TCGA-A3-3385	WT	6042324027_R05C01
TCGA-B0-5109	WT	6042324113_R01C02
TCGA-B0-5106	WT	6055424116_R05C01
TCGA-B0-4700	WT	6285617026_R06C01
TCGA-B8-5546	WT	6285617068_R03C02
TCGA-B0-5691	WT	6285617068_R04C01
TCGA-B2-5635	WT	6929671134_R04C02
TCGA-CJ-5680	WT	6929671134_R06C01
TCGA-DV-5576	WT	6929671144_R04C01
TCGA-EU-5905	WT	6929718088_R03C01
TCGA-B8-5545	WT	6929718090_R02C01
TCGA-CW-6087	WT	6929718091_R06C01
TCGA-B2-5635	WT	9305216018_R05C01
TCGA-B2-5635	WT	9305216018_R06C01
TCGA-CZ-5459	MUT	6042308132_R01C02
TCGA-B0-4710	MUT	6042308132_R03C02
TCGA-BP-5198	MUT	6042308144_R02C02
TCGA-BP-5178	MUT	6042308152_R05C01
TCGA-B0-5402	MUT	6042308163_R02C01
TCGA-B0-4852	MUT	6042308163_R05C01
TCGA-BP-5169	MUT	6042308164_R06C01
TCGA-CZ-5470	MUT	6042316009_R03C01
TCGA-B0-4712	MUT	6042316009_R06C02
TCGA-B0-4811	MUT	6042316038_R02C02
TCGA-B0-5399	MUT	6042316038_R05C01
TCGA-CZ-5461	MUT	6042316038_R06C01
TCGA-B0-4814	MUT	6042316057_R02C01

TCGA-B0-4822	MUT	6042324004_R04C02
TCGA-B0-4703	MUT	6042324004_R05C02
TCGA-B0-4718	MUT	6042324009_R03C02
TCGA-AK-3431	MUT	6042324009_R04C01
TCGA-A3-3367	MUT	6042324022_R06C01
TCGA-B0-4845	MUT	6042324033_R01C01
TCGA-B0-5121	MUT	6042324113_R05C02
TCGA-B0-5699	MUT	6285617026_R04C01
TCGA-CJ-5682	MUT	6285617026_R05C02
TCGA-A3-3358	MUT	6285617068_R05C01
TCGA-CJ-5676	MUT	6285650093_R03C02
TCGA-CJ-5684	MUT	6285650095_R02C02
TCGA-CW-5591	MUT	6285650095_R03C01
TCGA-CJ-5678	MUT	6285650095_R06C01
TCGA-CJ-5671	MUT	6929671144_R01C01
TCGA-CW-5580	MUT	6929718092_R05C01

Table S3. TCGA samples used from KIRP dataset

TCGA ID	SETD2 status	450K chip and location
TCGA-5P-A9KA	WT	3999510144_R03C02
TCGA-EV-5903	WT	6285609019_R05C01
TCGA-DW-5561	WT	6285609045_R02C01

TCGA-DW-7838	WT	7310440061_R05C02
TCGA-GL-7966	WT	7786923102_R02C02
TCGA-PJ-A5Z9	WT	9296931048_R06C02
TCGA-GL-A59T	WT	9296931055_R03C02
TCGA-Q2-A5QZ	WT	9296931055_R04C01
TCGA-IZ-A6M9	WT	9305216028_R04C02
TCGA-MH-A55W	WT	9305216203_R04C01
TCGA-SX-A7SQ	WT	9630789214_R06C02
TCGA-SX-A71U	WT	9630789230_R01C01
TCGA-IA-A83S	WT	9630789230_R06C01
TCGA-PJ-A8JU	WT	9630789235_R04C01
TCGA-BQ-5877	MUT	6285609045_R04C02
TCGA-HE-7130	MUT	6285633034_R02C01
TCGA-DZ-6133	MUT	6285633034_R03C02
TCGA-BQ-7061	MUT	6285633053_R02C01
TCGA-BQ-5891	MUT	6929671163_R02C02
TCGA-A4-7583	MUT	7310440066_R05C01
TCGA-A4-A5Y1	MUT	9296931055_R06C01
TCGA-BQ-5875	MUT	6285609019_R04C02
TCGA-A4-8098	MUT	7800246206_R03C02
TCGA-B9-5155	MUT	6285609019_R05C02
TCGA-G7-6793	MUT	6285633053_R05C02
TCGA-BQ-5876	MUT	6929689007_R02C01
TCGA-B9-A44B	MUT	8784225052_R03C01
TCGA-B1-A656	MUT	9305216028_R05C02
TCGA-BQ-7058	MUT	6285633053_R02C02

Table S4. TCGA samples used from LuCa dataset

TCGA ID	SETD2 status	450K chip and location
TCGA-71-6725	WT	6285625070_R03C02
TCGA-55-7726	WT	6285633063_R04C01
TCGA-49-6761	WT	6929671144_R05C02
TCGA-44-6774	WT	6285625076_R01C01
TCGA-MP-A4SV	WT	8784241084_R03C01
TCGA-L4-A4E6	WT	8795194077_R02C02
TCGA-44-3917	WT	9305216009_R03C01
TCGA-99-8028	WT	6264488026_R04C01
TCGA-50-5931	WT	6285625064_R06C02
TCGA-55-6975	WT	6285650110_R02C02
TCGA-44-A47G	WT	8795194077_R01C01
TCGA-86-8054	WT	6264488067_R02C02
TCGA-55-8089	WT	6264488067_R04C01
TCGA-55-7227	WT	6929742041_R01C01
TCGA-64-5775	MUT	6042308138_R01C01
TCGA-50-5942	MUT	6264488083_R02C02
TCGA-49-4487	MUT	6285625070_R05C02
TCGA-05-4396	MUT	6285625070_R06C01
TCGA-67-6216	MUT	6285625087_R03C02
TCGA-38-4631	MUT	6285625090_R05C02
TCGA-75-5126	MUT	6285625099_R01C01
TCGA-73-4658	MUT	6285625064_R05C02
TCGA-50-5941	MUT	6285625064_R06C01
TCGA-05-4427	MUT	6929671148_R04C02
TCGA-49-6744	MUT	6929671163_R01C01
TCGA-73-4676	MUT	6264488083_R06C02
TCGA-05-4425	MUT	6285625064_R04C01
TCGA-44-2666	MUT	9305216007_R05C02

Table S5. Database and Accession numbers for datasets used in study.

Database	Accession Number	Dataset Description
GEO	GSE66884	ChIP-seq for H3K36me3 for 786-O cells
GEO	GSE66879	RNA-seq for 786-O cells
GEO	GSM670025	Human Adult Kidney H3K4me1
GEO	GSM1112806	Human Adult Kidney H3K27ac
ENCODE	ENCSR071ZMO	Human Adult Kidney RNA-seq
GEO	GSM910572	Human Adult Lung H3K4me1
GEO	GSM906395	Human Adult Lung H3K27ac

SUPPLEMENTAL EXPERIMENTAL PROCEDURES

ChIP-qPCR

786-O parental cells and SETD2 KO1 and KO2 isogenic derivatives were grown in 150 mm cell culture dishes and 4 X 10^6 cells/ChIP reaction were used. When the cells reached ~80% confluency, plates were removed from the incubator and left at room temperature for 10 minutes. Next, 0.27 mL of 37% paraformaldehyde was add to the plate with 10 mL of media and incubated at room temperature for 10 minutes with swirling followed by the addition of 0.5 mL of 2.5 M glycine and incubation for an additional five minutes with swirling to quench the paraformaldehyde. Media was removed and 10 mL of cold TBS buffer was added to the cells, and then the cells were scraped and collected into a 15 mL tube. Cells were centrifuged at 500 RPM for 10 minutes. Supernatant was removed and 1 mL of cell lysis buffer (10 mM Tris-HCl, pH 7.5, 10 mM NaCl, 0.5% NP-40) was added per 4 X 10^6 cells. The cells were vortexed and incubated on ice for 10 minutes. Cells were then centrifuged at 3,000 RPM for five minutes at 4ºC. The supernatant was removed, and 0.5 mL of MNase digestion buffer was added (20 mM Tris-HCl, pH 7.5, 15 mM NaCl, 60 mM KCl, 1 mM $CaCl_2$) and the sample was briefly vortexed. The sample was centrifuged again at 3,000 RPM for five minutes at 4ºC, the supernatant was removed, and 0.5 mL of MNase digestion buffer was added. The sample was mixed well, and transferred to a 1.5 mL tube. MNase enzyme (New England Biolabs) was diluted 1:10 with MNase digestion buffer. Five µl of 10X diluted MNase was added to the sample, and the sample was then incubated at 37ºC for 20 minutes at 1,000 RPM shaking in a thermomixer. In this condition, at least 80-90% of the chromatin is digested into mono- and di- nucleosomes. 500 µl of Stop/ChIP buffer (100 mM Tris-HCl, pH 8.0, 20 mM EDTA, 200 mM NaCl, 2% Triton X-100, 0.2% sodium deoxycholate) was added and the sample was briefly vortexed. Samples were then sonicated in a Diagenode Bioruptor (15 cycles in Stop/ChIP buffer: 30 seconds on, 30 seconds off). The sample was then centrifuged at 15,000 RPM for 10 minutes at 4ºC and then transferred to a new tube. Chromatin concentration was determined using the Qubit system per

the manufacturer protocol. 20 µg of chromatin from each cell-type was used for H3K36me3 ChIP and 14.6 µg was used for the IgG control ChIP reaction. 1% of the sample was saved as the input for downstream real-time qPCR enrichment reactions. 2 µg of antibody for IgG (Abcam 37355) and H3K36me3 (Active Motif 61021) was added to the chromatin and rotated overnight at 4ºC. Magnetic protein-G agarose beads (Dynabeads) were pre-washed, and 30 µl were added to each sample. Samples were rotated at 4ºC for three hours. Using a magnetic rack the beads were washed as follows: 1 mL of 1X ChIP buffer, 1 mL of 1X ChIP buffer followed by five minute rotation at 4ºC, 1 mL of high salt buffer (ChIP buffer with 0.5 M NaCl), 1 mL of high salt buffer followed by five minute rotation at 4ºC, 1 mL of Tris/LiCl buffer (10 mM Tris-HCl pH 8.0, 0.25 M $LiCl_2$, 0.5% NP-40, 0.5% sodium deoxycholate, 1 mM EDTA), 1 mL of Tris/LiCl buffer followed by five minute rotation at 4ºC, 1 mL of TE buffer twice. To elute the captured chromatin from the beads, 50 µl of 1X elution buffer (10 mM Tris-HCl pH 8.0, 10 mM EDTA, 150 mM NaCl, 5 mM DTT, 1% SDS) was added to the beads and incubated at 65ºC for 15 minutes. During this incubation the sample was vortexed for five seconds every three minutes. The elution was repeated a second time and combined with the previous collection. Eluents were incubated overnight at 65ºC to reverse cross-link the DNA. At this point, the input is brought to the same volume as the ChIP sample with elution buffer and processed alongside the eluent. The next day, the sample and input were treated with RNase A (2 µg) for one hour at 37ºC. 10 µl of 20 mg/mL proteinase K was added to the sample and incubated at 37ºC for two hours. The DNA samples were then purified with the Qiagen MinElute kit per the manufacturer's protocol with elution in 10 µl of elution buffer. Samples were stored at 4ºC prior to real-time qPCR analysis for enrichment. Enrichment analysis was performed with qPCR in triplicate reactions. The pull-down sample was normalized to the 10% input by subtracting the C(t) value of the input from the sample ($\Delta C(t) = C(t)_{sample} - C(t)_{input}$). Next, the relative enrichment was calculated (relative enrichment = $2^{-\Delta C(t)}$).

ChIP-seq data analysis

Raw sequencing reads were mapped to the UCSC human genome hg19 build using BWA V0.5.9 [2] with a default parameter setting. Multiply mapped reads and uniquely mapped reads with mismatches and indels > 5% of read lengths were filtered out. SICER V1.1 [3] was used to identify peaks in a sample and differentially enriched regions between two samples relative to an input with the following parameters: redundancy allowed = 1, window size = 200, fragment size = 300, effective genome size = 0.833, gap size = 400, E-value = 1000, false discovery rate = 0.01. In-house scripts annotated peaks and differentially enriched regions with RefSeq, CGIs, and repeats in the UCSC genome browser [4], and classified them as promoter (−1kbp - +1 for TSS), body, and 3′ end (TTS + 1kbp). In some cases, gene bodies were further classified into 5′ UTR, exon, protein coding exon, 3′ UTR, and intron. After discarding more than two reads mapping to the same location, mapped reads were lengthened to the 3′-end to reflect their original length, and counted based on their midpoint for genomic features such as genes, CGIs, and repeats. A genomic feature was binned by relative positions including upstream and downstream regions. Different numbers of mapped reads per sample were taken into account by calculating FPKM (fragments per kilobase per million fragments mapped). To illustrate the change in tag densities around genes, a relative length window for gene bodies was used and the average of normalized read coverage in a window was measured. For integration with 450K array datasets, bedtools was used to determine intersection between ChIP-seq peaks and CpG loci [5].

450K array data analysis

Quality control of 450K array samples was assessed using the Genome Studio Methylation Module (Illumina). Subset-quantile Within Array Normalization (SWAN) was performed on IDAT files via the R Bioconductor package “minfi” [6] and annotated to genome build h19. Probes with a detection pval ≥ 0.05 were eliminated from the analysis. Probes from the X and Y chromosome were retained for downstream analysis as both 786-O and NCCIT cell lines are

derived from males. Sex chromosomes were removed from analysis of TCGA datasets due to genetic heterogeneity. CpGs were considered differentially methylated for 786-O isogenic cell line models if $|\Delta\beta| \geq 0.2$ as long-term KO of SETD2 induced substantial changes in DNA methylation, and for NCCIT siKD cell line and TCGA KIRC set $|\Delta\beta| \geq 0.1$ as the magnitude of differential methylation was lower compared to the knockout model. Spatial distribution plots for 450K array data were generated using in-house scripts. Normalization for the genomic feature enrichment plots was conducted by comparing the number of significantly differentially methylated CpG sites to the total number of sites in each genomic feature present on the 450K array. Clustering and heatmaps were performed in the R packages “heatmap” and “heatmap.3” or using Microsoft Excel. Heatmaps for average methylation and change in methylation across a gene was conducted by averaging the Avgβ and Δβ-value of CpGs on the 450K array assigned to particular genes. Unsupervised hierarchical clustering in Figures 7G-I was performed using the top 10,000 most variable CpGs. PanCancer analysis in Figure 8A was done with supervised clustering by obtaining a signature conserved in all three tumor types. The significance of all clustering (SETD2 mutant vs WT and clinical staging) was calculated by chi-square tests.

Supplemental References

1. Ehrlich M, Woods CB, Yu MC, Dubeau L, Yang F, Campan M, Weisenberger DJ, Long TI, Youn B, Fiala ES and Laird PW. Quantitative analysis of associations between DNA hypermethylation, hypomethylation, and DNMT RNA levels in ovarian tumors. Oncogene. 2006; 25(18):2636-2645.
2. Li H and Durbin R. Fast and accurate short read alignment with Burrows–Wheeler transform. Bioinformatics. 2009; 25(14):1754-1760.
3. Zang C, Schones DE, Zeng C, Cui K, Zhao K and Peng W. A clustering approach for identification of enriched domains from histone modification ChIP-Seq data. Bioinformatics. 2009; 25(15):1952-1958.
4. Fujita PA, Rhead B, Zweig AS, Hinrichs AS, Karolchik D, Cline MS, Goldman M, Barber GP, Clawson H, Coelho A, Diekhans M, Dreszer TR, Giardine BM, Harte RA, Hillman-Jackson J, Hsu F, et al. The UCSC Genome Browser database: update 2011. Nucleic Acids Res. 2011; 39(suppl 1):D876-D882.
5. Quinlan AR and Hall IM. BEDTools: a flexible suite of utilities for comparing genomic features. Bioinformatics. 2010; 26(6):841-842.
6. Maksimovic J, Gordon L and Oshlack A. SWAN: Subset-quantile Within Array Normalization for Illumina Infinium HumanMethylation450 BeadChips. Genome Biol. 2012; 13(6):R44.
